# Geometric morphometric analysis of Pleuronectiformes vertebrae: A new tool to identify archaeological fish remains?

**DOI:** 10.1111/joa.13934

**Published:** 2023-07-26

**Authors:** Katrien Dierickx, Tarek Oueslati, Antonio Profico

**Affiliations:** ^1^ Department of Archaeology University of York York UK; ^2^ Department of Archaeology and Cultural History NTNU University Museum Trondheim Norway; ^3^ Centre National de la Recherche Scientifique University of Lille Lille France; ^4^ Department of Biology University of Pisa Pisa Italy

**Keywords:** fish remains, geometric morphometrics, ichthyoarchaeology, zooarchaeology

## Abstract

Flatfish (Pleuronectiformes) vertebrae are difficult to identify to species due to the lack of diagnostic features. This has resulted in a lack of understanding of the species abundances across archaeological sites, hindering interpretations of historical fisheries in the North Sea area. We use a new approach, utilising a combined 2D landmark‐based geometric morphometric analysis as an objective and non‐destructive method for species identification of flatfish vertebrae from the North Sea area. Modern specimens were used as a reference to describe the morphological variation between taxa using principal component analysis (PCA) and to trial an automated classification using linear discriminant analysis. Although there is limited distinction between taxa using PCAs, the classification shows high accuracies, indicating that flatfish species identifications using geometric morphometrics are possible. Bone samples (*n* = 105) from two archaeological sites in the United Kingdom and France were analysed using this approach and their identifications were verified using collagen peptide mass fingerprinting. The success rate of species identification was usually less than 50%, indicating that this technique has limited applicability due to preservation/fragmentation of archaeological fish bone. Nonetheless, this could prove a valuable tool for modern and non‐fragmented samples. Furthermore, the technique applied in this study can be easily adapted to work on other landmark datasets.

## Graphical abstract

Flatfish vertebrae were analysed using a combined 2D landmark‐based geometric morphometric analysis to identify the species of archaeological remains. Although good morphological distinction and identification of modern and complete vertebrae is possible, this method has limited applicability on fragmented archaeological material, as indicated by collagen peptide mass fingerprinting.

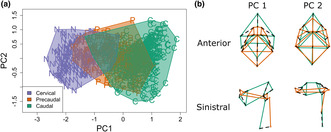

## 1 INTRODUCTION

Studying flatfish bones from archaeological sites around the North Sea area can help to better understand shifts in the environment, economy, fisheries and human diet throughout history. There are currently over 20 species of Pleuronectiformes reported from the North Sea (Heessen et al., 2015). Of all flatfish species in the North Sea *Platichthys flesus* (Linnaeus 1758) (flounder) and *Pleuronectes platessa* Linnaeus 1758 (plaice) are very similar in morphology and rarely get confidently identified to species based on a single vertebra from archaeological remains. It is, however, of great interest to correctly identify these two species, since they are the most commonly reported species from archaeological sites around the North Sea and have been of economic importance for this area for centuries (e.g. Harland et al., 2016; Locker, 2007; Oueslati, 2019; Reynolds, 2015; Van Neer & Ervynck, 2007). Differentiating between species that can occur in freshwater environments, such as *P. flesus*, from the marine species could be useful to better understand changes in fisheries through time. Also identifying species that are known from the more northern or southern areas from the North Sea, such as for example *Hippoglossus hippoglossus* (Linnaeus 1758) and *Solea solea* (Linnaeus 1758) respectively, can help to uncover environmental changes in the North Sea as well as potentially expose the trade of fish (Ervynck et al., 2004).

Archaeological studies on disarticulated fish remains from the North Sea in Europe often show that many flatfish (Pleuronectiformes) bones can be difficult to identify at species level, although they are very commonly recovered from these sites. This difficulty resulted in many skeletal elements remaining unidentified or only being identified to a higher taxonomic level (e.g. Enghoff, 1999; Ervynck & Van Neer, 1992; Harland et al., 2016; Nicholson, 2009; Oueslati, 2019; Reynolds, 2015; Wouters et al., 2007). This issue is especially true for vertebrae of flatfish. Vertebrae are difficult to use to identify species due to the lack of clear diagnostic criteria between species (e.g. Clavel, 1997; Wouters et al., 2007). To date, there has been no in‐depth study of flatfish vertebrae shape and how it can be used to identify species. Only a few publications provide descriptions of vertebra morphology of some flatfish species found in the North Sea (e.g. Watt et al., 1997; Wouters et al., 2007).

Geometric morphometrics (GMM) is a landmark‐based morphometric approach to analyse and compare the shape and form of objects by comparing the relative position of landmarks. It is often used to analyse shape variations within an evolutionary context (e.g. Black & Berendzen, 2020), to aid in taxon identification (e.g. Santos et al., 2019) and can even distinguish between populations (e.g. Ibañez et al., 2007). Some studies have applied GMM on scutes, scales and otoliths of other fish groups, indicating the possibility to use these skeletal elements for species identifications (e.g. Ibañez et al., 2007; Ponton, 2006; Thieren & Van Neer, 2014). No study of flatfish vertebrae using GMM has been performed so far, and the number of studies applying this technique on vertebrae of other fish taxa is also limited. Guillaud et al. (2016) used three to seven landmarks to identify modern and archaeological Salmonidae vertebrae to species relatively easily. GMM has also been applied to identify the habitat of archaeological fish remains by comparing the shape of the anterior and posterior sides of precaudal and caudal vertebrae of archaeological remains to a collection of samples from a known habitat (Samper Carro et al., 2018) and to estimate sizes (Dombrosky et al., 2022). These studies indicate the potential for this technique to differentiate flatfish by the shape of their vertebra.

In this paper, we measure the morphology of a modern and an archaeological sample collection of flatfish vertebrae by acquiring 2D pictures in anterior and left‐lateral (sinistral) views. In detail, we tested the following research questions: (i) does GMM determine flatfish vertebra type? (ii) is it possible to identify the taxonomic level of archaeological flatfish vertebrae by using a modern sample as a reference? We initially describe the morphological variation present in modern flatfish vertebrae using GMM. We then test the classification system by classifying modern flatfish vertebra to type and taxa using GMM on an ideal dataset before finally exploring the classification of archaeological flatfish vertebra to type and taxa using GMM to test the accuracy of species assignment.

## 2 MATERIALS AND METHODS

### 2.1 Sample selection

#### 2.1.1 Modern sample collection

Modern Pleuronectiformes specimens were selected from the fish bone collections housed at the Royal Belgian Institute of Natural Sciences (RBINS) and University of York (YZL). Seventy‐three flatfish from five different families and 19 species were sampled. Table 1 provides an overview of the species used and details can be found in Tables S1 and S2.

**TABLE 1:** Overview of the specimens used in this study. Details can be found in Table S1.

Family	Genus	Species	Author	Number of specimens	Remarks
Bothidae	*Arnoglossus*	*laterna*	(Walbaum 1792)	3	
Citharidae	*Citharus*	*linguatula*	(Linnaeus 1758)	2	
Pleuronectidae	*Glyptocephalus*	*cynoglossus*	(Linnaeus 1758)	5	
Pleuronectidae	*Hippoglossoides*	*platessoides*	(Fabricius 1780)	7	
Pleuronectidae	*Hippoglossus*	*hippoglossus*	(Linnaeus 1758)	5	
Pleuronectidae	*Limanda*	*limanda*	(Linnaeus 1758)	6	
Pleuronectidae	*Microstomus*	*kitt*	(Walbaum 1792)	7	
Pleuronectidae	*Platichthys*	*flesus*	(Linnaeus 1758)	6	Right‐eyed
Pleuronectidae	*Platichthys*	*flesus*	(Linnaeus 1758)	4	Left‐eyed
Pleuronectidae	*Pleuronectes*	*platessa*	Linnaeus 1758	6	
Scophthalmidae	*Lepidorhombus*	*boscii*	(Risso 1810)	1	
Scophthalmidae	*Lepidorhombus*	*whiffiagonis*	(Walbaum 1792)	3	
Scophthalmidae	*Scophthalmus*	*maximus*	(Linnaeus 1758)	4	
Scophthalmidae	*Scophthalmus*	*rhombus*	(Linnaeus 1758)	3	
Scophthalmidae	*Zeugopterus*	*punctatus*	(Bloch 1787)	1	
Scophthalmidae	*Zeugopterus*	*regius*	(Bonnaterre 1788)	1	
Soleidae	*Buglossidium*	*luteum*	(Risso 1810)	1	
Soleidae	*Dicologlossa*	*hexophthalma*	(Bennett 1831)	1	
Soleidae	*Pegusa*	*lascaris*	(Risso 1810)	2	
Soleidae	*Solea*	*solea*	(Linnaeus 1758)	5	

As *P. flesus* can be both left‐eyed and right‐eyed, this has an impact on the shape of the bones, especially the cranial bones, with both forms showing different characteristics in homologous bones. Both forms are in general each other's mirror image, although slight differences might be present (e.g. Wouters et al., 2007). This could mean that both forms could potentially also show slight shape differences in their vertebrae due to the asymmetry causing a mirror‐image effect, which could be detected using GMM.

#### 2.1.2 Archaeological sample collection

**FIGURE 1:**
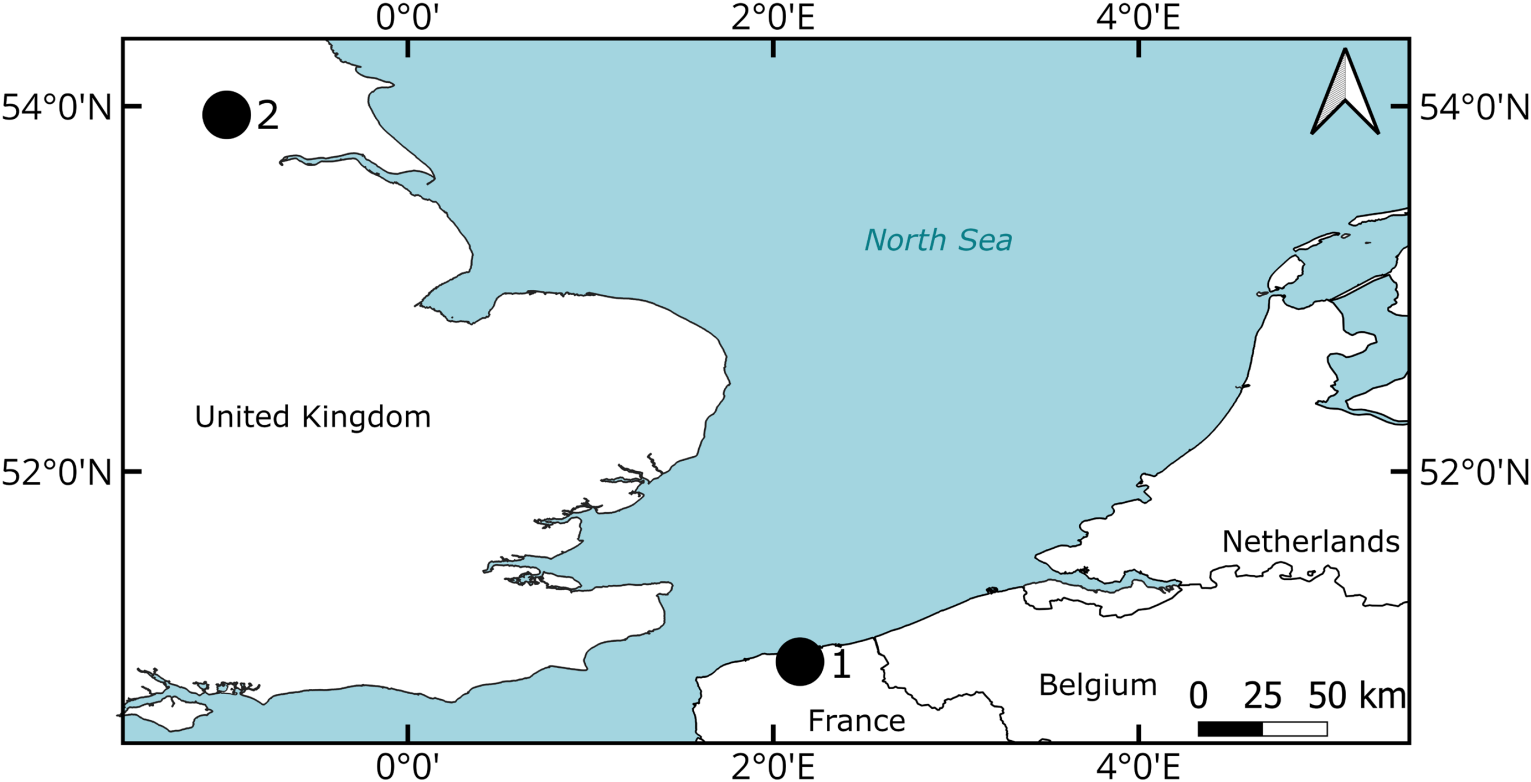
Map with the locations of the two analysed archaeological sites. 1: Barreau Saint‐George‐Desserte ferroviaire; 2: 16–22 Coppergate.

A total of 105 archaeological samples were analysed from two archaeological sites (Figure 1). Sixty‐one were derived from Barreau Saint‐George‐Desserte ferroviaire in northern France (50°58′27.8″ N, 2°10′7.6″ E) dating from the end of the 10th century to the beginning of the 11th century CE (Herbin & Oueslati, 2016). Most remains from this site were identified as Pleuronectidae and also a single *Solea solea* bone was uncovered (Oueslati, 2019). Forty‐four were sampled from 16 to 22 Coppergate in the United Kingdom (53°57′27.4″ N, 1°4′51.5″ W), a site in the walled city centre of York in northern England. This site dates from the Roman period (first to fourth century CE) to the Late Medieval period (13th–14th century CE). A large diversity of fish bones from many different families, including Pleuronectidae and Scophthalmidae, has been identified from this site (Harland et al., 2016).

The samples were identified morphologically as flatfish following diagnostic criteria published in Watt et al. (1997) and Wouters et al. (2007) and with comparison to modern reference specimens housed at the University of York. An overview of the samples can be found in Tables S4–S9.

### 2.2 Photography

Digital images were taken from the first vertebra or atlas, a few cervical vertebrae, a few precaudal vertebrae and some caudal vertebrae of every modern specimen. If possible, a minimum of 10 complete vertebrae per modern specimen were photographed. Modern and archaeological vertebrae were photographed from two views, anterior and sinistral. The dextral and posterior side were not included in this study as these do not provide much more morphological information than is not already available from the anterior and sinistral views. See Table S1 for details on the number of photos taken for each specimen.

For photography, a NIKON D5600 with an EX Sigma 105 mm 1:2.8 DG macro HSM lens with 62 mm diameter was used. The following settings of the camera were applied: ISO 200, F22, shutter 1″, manual mode, highest resolution JPEG, no zoom. The camera was mounted on an arm that could be moved to position the camera in an angle perpendicular to the surface on which the vertebrae were lying. The camera was placed at a distance of the bone to allow the whole vertebra, including the arches, to be in the image, usually 10–30 cm. A 10‐s timer allowed the camera to stabilise after pushing the shutter button, to get as sharp an image as possible. To support the vertebrae in the correct place, a piece of kneadable plasticine was used. By manually adjusting the lens, the image was focused on the structures important for landmarking. In every photo, a scale bar was placed at the same height as the focused structures, and a label indicating the species and collection number was added as well. A black, non‐shiny background, such as black cotton cloth, provided good contrast with the lightly coloured bones.

### 2.3 Landmark configurations

**FIGURE 2:**
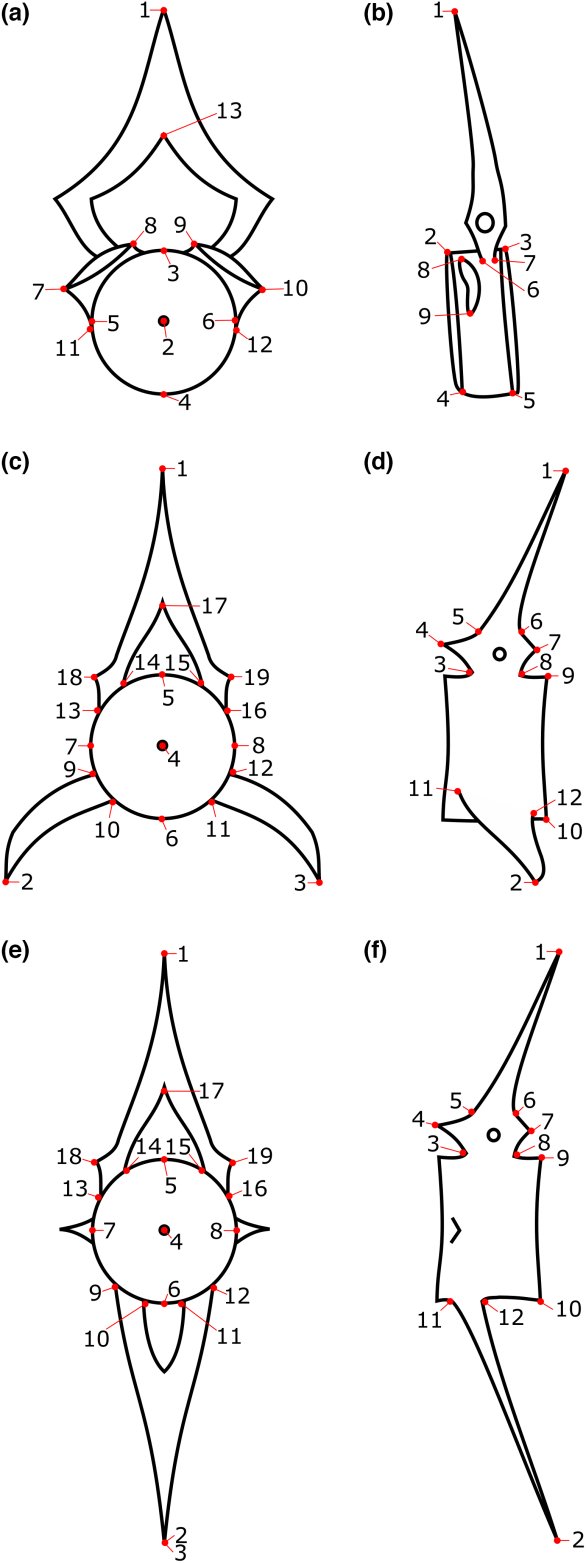
Landmark configurations. (a). Anterior view of atlas vertebra; (b). Sinistral view of atlas; (c). Anterior view of a precaudal vertebra; (d). Sinistral view of a precaudal vertebra; (e). Anterior view of caudal vertebra; (f). Sinistral view of a caudal vertebra. Numbers are explained in Table 2.

Landmark acquisition from photographs was carried out using TPSdig232 Version 2.31 (Rohlf, 2017). Landmarks of types 1, 2 and 3 were used (Bookstein, 1991). All landmarks were placed on predetermined structures present in all taxa as illustrated and described in Figure 2 and Table 2. Cervical, precaudal and caudal vertebrae were all landmarked in the same way using 19 landmarks in the anterior view and 12 in sinistral view. Cervical vertebrae have fewer landmarks available (see LM with an * in Table 2) and these were labelled as missing landmarks using TPSdig232. Atlas vertebrae followed a different landmark configuration with 13 landmarks in anterior view and nine in sinistral view. Using the scale bar on the photographs, all photographs could be scaled, using 1 cm. All TPS files received a unique name, with their order number, sample identifier, type of vertebra, view, family, genus and species code.

**TABLE 2:** Definition of landmarks used per view and vertebra type, that is, atlas and other (cervical, precaudal and caudal). Landmarks indicated with “a” are absent in cervical vertebrae, but present in precaudal and caudal vertebrae.

No.	Anterior atlas	Anterior other	Sinistral atlas	Sinistral other
1	Tip of neural arch	Tip of neural arch	Tip of neural arch	Tip of neural arch
2	Notochord of centre	Tip of dextral haemal arch^a^	Dorsal edge of anterior side of centre	Tip of haemal arch or sinistral haemal processus
3	Most dorsal part of the edge of the centre	Tip of sinistral haemal arch^a^	Dorsal edge of posterior side of centre	Ventral implant of sinistral anterior dorsal spina
4	Most ventral part of the edge of the centre	Notochord of centre	Ventral most part of anterior part of centre	Tip of sinistral anterior dorsal spina
5	Dextral part of the edge of the centre, at the same height as LM4 and perpendicular to LM5–LM6	Most dorsal part of the edge of the centre	Ventral most part of posterior part of centre	Dorsal implant of sinistral anterior dorsal spina
6	Sinistral part of the edge of the centre, at the same height as LM4 and perpendicular to LM5–LM6	Most ventral part of the edge of the centre	Anterior implant of neural arch	Dorsal implant of sinistral posterior dorsal spina
7	Lateral edge of dextral condylus	Dextral part of the edge of the centre, at the same height as LM4 and perpendicular to LM5–LM6	Posterior implant of neural arch	Tip of sinistral posterior dorsal spina
8	Medial edge of dextral condylus	Sinistral part of the edge of the centre, at the same height as LM4 and perpendicular to LM5–LM6	Dorsal most edge of condylus	Ventral implant of sinistral posterior dorsal spina
9	Medial edge of sinistral condylus	Lateral implant of the dextral haemal processus^a^	Ventral most edge of condylus	Dorsal edge of posterior side of centre
10	Lateral edge of sinistral condylus	Medial implant of the dextral haemal processus^a^		Ventral edge of posterior side of centre
11	Lateral implant at centre of dextral condylus	Medial implant of the sinistral haemal processus^a^		Anterior implant of haemal arch or sinistral haemal processus^a^
12	Lateral implant at centre of sinistral condylus	Lateral implant of the sinistral haemal processus^a^		Posterior implant of haemal arch or sinistral haemal processus^a^
13	Dorsal most part of the neural canal	Lateral implant of the dextral neural processus		
14		Medial implant of the dextral neural processus		
15		Medial implant of the sinistral neural processus		
16		Lateral implant of the sinistral neural processus		
17		Dorsal most part of the neural canal		
18		Dorsal tip of the dextral anterior dorsal spina		
19		Dorsal tip of the sinistral anterior dorsal spina		

As the neural and haemal arches are often absent in archaeological samples due to the preservation (see Table S10), the landmarks on the tips of these arches (LM 1 in atlas anterior and sinistral views, LM 1, 2 and 3 in the other vertebrae anterior view, and LM 1 and 2 in the other vertebrae sinistral view) are not further included in the analysis. PCA plots of the analyses with and without these landmarks show minor differences in distinction of the taxa (Figures S21–S24).

### 2.4 Analysis

#### 2.4.1 Description of shape variation in modern samples

TPS files were analysed with R (R Core Team (2022), version 4.1.1 (2021‐08‐10)—‘Kick Things’) using the following packages: MASS, caret (Kuhn, 2008), geomorph (Adams & Otárola‐Castillo, 2013), Arothron (Profico et al., 2017) and Morpho (Schlager, 2017).

Two types of analyses were carried out, one set on the atlas vertebrae (*n* = 69), and the other set on the cervical, precaudal and caudal vertebrae (*n* = 1067) as these vertebra types differ in their morphological structures. Subsets were created to allow for particular comparisons between groups or taxa using specific sets of landmarks. Missing landmarks in the modern dataset were estimated using the *estimate.missing()* function in geomorph (also see Arbour & Brown, 2014). Any sample outside of the range provided by the interquartile range method was removed from the dataset.

The anterior and sinistral views were analysed by performing a PCA using the *procSym()* function. Afterwards, both views were analysed together using the *twodviews()* function in Arothron following Profico et al. (2019).

A plot of the principal component (PC) scores visualised the morphological variation among the samples. Negative and positive extreme variation along the first principal components have been used to produce shape variations by using *twodvarshape()* and *deformGrid2d()* functions from Arothron and Morpho packages respectively.

#### 2.4.2 Classification test using modern samples

A linear discriminant analysis (LDA) was performed to classify the specimens to vertebra types and taxonomic groups (family and species) and to assess the success rate of this classification. The analysis was run using all specimens to classify the vertebra types, families and species excluding LM 9–12 in the anterior view and LM 11 and 12 in the sinistral view. The analysis was also run for each vertebra type separately to classify families and species using the whole available landmark configuration for each vertebra type. Species with only one specimen were removed from the dataset for the analysis.

The LDA was performed 100 times for each subset of the modern dataset and the mean of the accuracy rate of all 100 runs was calculated. The analysis was performed on the anterior, sinistral and combined views. For each LDA, the modern samples were divided into two groups, a training set and a testing set, with a 70:30 ratio respectively. A GPA and PCA were performed using *procSym()* on the training set for individual views. To condense the data for ease of analysis and to reduce the computational time, a PCA using the *prcomp()* function on the PC scores of the first PCA was performed. The PC scores from this second PCA were taken to create a training model with the *train()* function using an LDA. The testing set was then standardised using the mean shape of the first PCA of the training set, as the testing set is proportionally large and would otherwise influence the mean shape of both sets combined. Using the *predict()* function the landmark data after standardisation of the testing set is converted into PC scores. Using these PC scores the vertebra type or taxonomy of each sample is calculated using the LDA training model. Based on the specimen data, the accuracy of the classification was then determined. To analyse and classify using the combined view, the PC scores of the training dataset of the individual views using *procSym()* were combined into one dataframe to create the training model. The landmark configurations of the testing sets of the individual views were transformed individually using the corresponding mean shape of the first PCAs for the individual views and were combined afterwards to convert the landmark data into PC scores of the testing sets to classify the samples.

#### 2.4.3 Identification of archaeological samples

Each archaeological sample was analysed individually against the modern reference dataset using LDA to try to identify the most probable vertebra type, family and species.

Landmarks that were not present in the archaeological sample, were also removed from the reference dataset. A GPA and PCA were performed using *procSym()* combining the archaeological sample and a selected subset of the reference dataset for individual views. The subset was determined by the identification level required. The first step was to identify the vertebra type of each sample, for which all cervical, precaudal and caudal reference samples were included, though using the reduced landmark configuration, excluding LM 9–12 in the anterior view and LM 11 and 12 in the sinistral view. From this subset, the most probable family could be identified, after which only the reference samples from this most probable family were used to identify the most probable species. The analysis was also run with the vertebra type given, as this could be identified visually, to identify the family and subsequently the species of each archaeological sample. This was also the approach used for the atlas vertebrae.

To condense the data for ease of analysis and to reduce the computational time, a PCA using the *prcomp()* function on the PC scores of the first PCA was performed using only those of the reference subset. The landmark configuration of the archaeological sample did not need to be standardised using the mean shape of the first PCA of the reference set as the subset consists mostly of the reference samples and only one archaeological sample, causing the mean shape to be based mostly on the data from the reference subset. The PC scores of the archaeological sample after the initial GPA and PCA were used to calculate the vertebra type or taxonomy using the LDA training model. To analyse and classify using the combined view, the PC scores of the reference dataset of the individual views were combined into one dataframe to create the training model. The PC scores for the combined view of the archaeological sample were obtained by using the *twodviews()* function.

For each sample the most probable family and species were noted as well as the probability score for the classification. The accuracy of GMM on archaeological samples was confirmed by identifying the samples using collagen peptide mass fingerprinting (ZooMS), following Dierickx et al. (2022), where the results of the identifications of the archaeological samples of this study were published.

**FIGURE 3:**
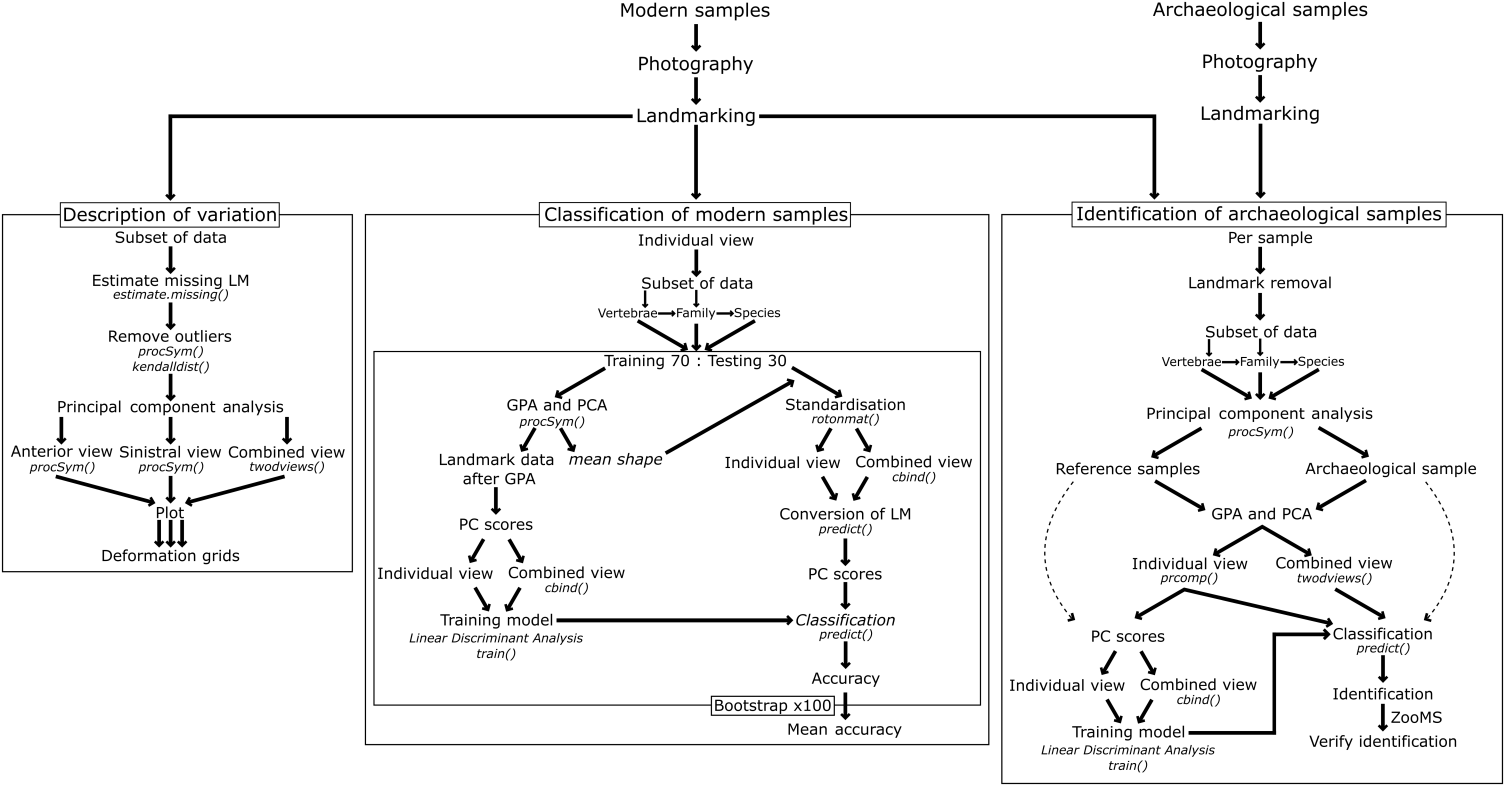
Workflow of methodology.

Figure 3 provides a schematic workflow of the analysis.

## 3 RESULTS

### 3.1 Morphological shape variation in modern samples

The variation of the modern samples is described using PCA (Figures 4 and 5, Table 3 and Figures S1–S25) by using GMM. Below only vertebra type and family level distinctions are detailed and visualised. Details of species level distinctions can be found in the supplementary information.

**FIGURE 4:**
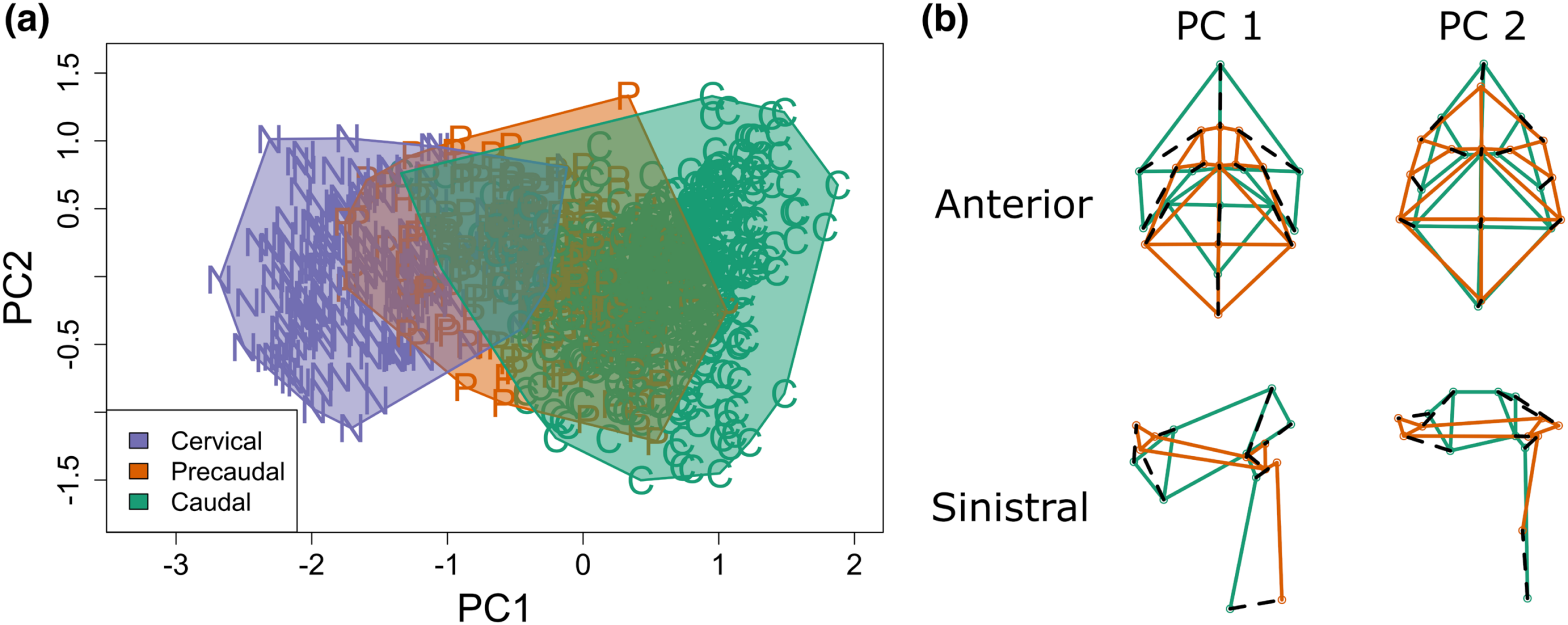
(a). Scatterplot of PC1 against PC2 for a principal component analysis of all cervical, precaudal and caudal samples (*n* = 1067) grouped by vertebra type using the combined view; (b). Deformation grids of anterior (upper) and sinistral (lower) views for both PC1 (left) and PC2 (right) comparing the minimal deformation (green) with the maximum deformation (red).

**FIGURE 5:**
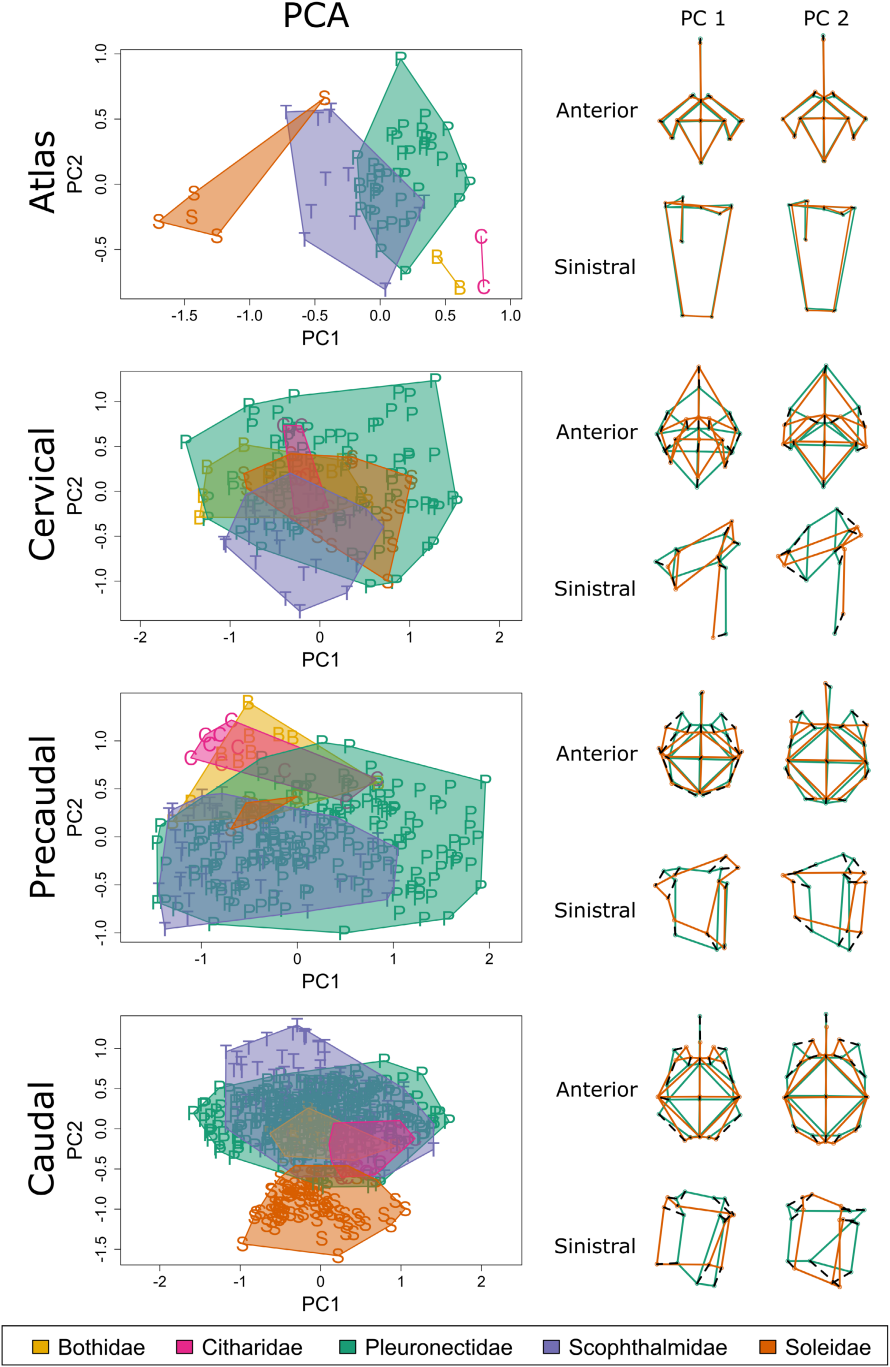
Principal component analysis plots and shape variation plots of PC1 and PC2 for combined view for each vertebra type; convex hulls by family level. Minimal deformation of the shape variation is green and the maximum deformation is red.

**TABLE 3:** PCA variance explained by the first three PC axes for different subsets and vertebra types.

Vertebra type	Group	Combined view			Anterior view			Sinistral view		
		PC1	PC2	PC3	PC1	PC2	PC3	PC1	PC2	PC3
All	Vertebra type	49.12	12.7	5.74	64.92	9.93	6.59	39.49	25.33	7.99
Atlas	Family	25.04	13.98	11.68	32.17	20.84	14.98	20.38	19.75	17.81
Atlas	Pleuronectidae	21.01	15.87	11.54	24.22	19.54	14.54	26.7	23.26	17.38
Atlas	Scophthalmidae	43.24	16.41	13.34	51.63	18.78	10.42	39.79	28.44	15.16
Atlas	Soleidae	53.45	22.7	9.85	61.68	19.46	11.27	54.4	24.87	8.86
Atlas	*P. platessa/P. flesus*	24.74	21.88	13.52	38.22	19.34	15.68	35.02	20.23	14.76
Cervical	Family	27.25	14.92	10.21	44.92	16.43	10.66	29.56	22.07	13.84
Cervical	Pleuronectidae	29.5	15.24	12.87	50.78	17.64	7.36	32.83	25.35	12.37
Cervical	Scophthalmidae	30.58	20.59	11.37	48.05	18.53	10.26	40.98	18.27	10.78
Cervical	Soleidae	36.66	17.44	12.09	45.57	19.42	13.33	41.42	25.54	11.87
Cervical	*P. platessa/P. flesus*	31.29	18.64	13.1	46.13	18.65	12.66	45.97	23.01	12.88
Precaudal	Family	40.12	11.53	8.53	52.2	13.99	7.7	31.67	21.87	10.46
Precaudal	Pleuronectidae	43.57	11.31	7.64	54.15	11.96	8.45	35.4	21.2	10.97
Precaudal	Scophthalmidae	43.83	17.75	6.95	61.5	10.72	8.05	39.19	23.6	9.5
Precaudal	Soleidae	46.31	26.81	16.73	48.17	29.41	14.49	45.32	25.43	22.22
Precaudal	*P. platessa/P. flesus*	36.66	11.85	8.33	43.69	14.75	8.78	34.76	16.3	12.1
Caudal	Family	30.18	20.68	10.32	46.02	13.7	8.84	40.72	22.72	11.21
Caudal	Pleuronectidae	39.1	12.04	8.68	47.96	12.88	8.06	42.18	15.07	13.84
Caudal	Scophthalmidae	37.65	22.75	7.92	46.73	18.73	9.86	48.33	23.65	6.22
Caudal	Soleidae	43.79	12.23	7.14	52.12	10.38	9.44	51.73	13.93	9.17
Caudal	*P. platessa/P. flesus*	44.51	10.71	9.43	54.98	10.23	7.74	46.03	18.79	11.58

PCA on the entire modern dataset, excluding the atlas vertebrae, has been performed by selecting only anatomical landmarks shared by different vertebra types (LM 4–8 and 13–19 anterior view and 3–10 sinistral view). The first two PC scores are associated with 61.82% of the total variance (Table 3). PC1 describes variations among vertebra types. Cervical vertebrae are at negative values of PC1, precaudal vertebrae are placed around neutral values and caudal vertebrae at positive values of PC1. PC2 variations are related to variability within vertebra morphology. Shape variations at the extreme values of PC1 are associated with the relative depth of the vertebra and the size and implantation or the neural arch base. Shape variations at the extreme values of PC2 are associated with the relative depth of the vertebra.

Using only the atlas vertebrae, the first two PC scores are associated with 39.02% of the total variance (Table 3). PC1 and PC2 describe variations between the five families, which are well separated (Figure 5 and Figure S1). Shape variations at the extreme values of PC1 are associated with the relative size and shape of the condyles. Shape variations at the extreme values of PC2 are associated with the shape of the centre. Within Pleuronectidae, the species seem to be slightly separated (Figure S2). *Pleuronectes platessa* and *P. flesus* can be slightly separated from each other on PC2 and PC3 (Figures S2 and S3). Scophthalmidae species separate strongly in PC1 and PC2 (Figure S4). Soleidae species separate clearly (Figure S5), but there are only a few specimens.

Using the cervical vertebrae (Figure 5 and Figure S6), the first two PC scores are associated with 42.17% of the total variance (Table 3). PC1 and PC2 describe variations between the families, which are not separated. Shape variations at the extreme values of PC1 are associated with the relative size and shape of the centre and the base of the neural arch. Shape variations at the extreme values of PC2 are associated with the size and implantation of the neural arch base. Pleuronectidae (Figures S7 and S8) and Soleidae (Figure S10) species also show no distinction. Within Scophthalmidae there seems to be a slight separation on PC2 and PC3 in combined view (Figure S9).

Using the precaudal vertebrae (Figure 5 and S11), the first two PC scores are associated with 51.65% of the total variance (Table 3). PC1 and PC2 describe variations between the families, which show little separation. Pleuronectidae and Scophthalmidae overlap. Shape variations at the extreme values of PC1 are associated with the relative size and shape of the base of the neural arch. Shape variations at the extreme values of PC2 are associated with the relative depth of the centre. Within Pleuronectidae there are slight differences noticeable between the species, but all species still overlap largely (Figure S12). *Pleuronectes platessa* and *P. flesus* differ on PC2 and PC3 in the combined view (Figure S13). Within Scophthalmidae there is a clear separation between the species on PC2 and PC3 (Figure S14).

Using the caudal vertebrae (Figure 5 and Figure S16), the first two PC scores are associated with 50.86% of the total variance (Table 3). PC1 and PC2 describe variations between the families, which are slightly separated. Shape variations at the extreme values of PC1 are associated with the implantation of the haemal arches and relative depth of the centre. Shape variations at the extreme values of PC2 are associated with the relative depth of the centre and the distance between the neural arch bases. Within Pleuronectidae, there is a slight separation between the species on PC2 and PC3 (Figure S17). *Pleuronectes platessa* and *P. flesus* largely overlap (Figure S18). Within Scophthalmidae there is a separation between species on PC2 and PC3 (Figure S19). Within Soleidae the species largely overlap (Figure S20).

Between right‐eyed and left‐eyed *P. flesus* there seems to be a slight difference in morphology as seen in the anterior views in precaudal vertebrae along PC2 (Figure S25), but no clustering was observed in the other views or in the caudal vertebra. Atlas and cervical vertebrae were not analysed as there are only a handful of samples available.

### 3.2 Classification of modern samples

Five species were removed from the dataset when classifying modern samples, as these only had one specimen in the dataset with a limited number of TPS files: *Dicologlossa hexophthalma*, *Buglossidium luteum*, *Zeugopterus punctatus*, *Zeugopterus regius* and *Lepidorhombus boscii*. Citharidae and Bothidae were retained for family‐level classification, but as there was only one species in each of these families in the dataset, no further analysis was performed. The sample size was too small for the atlas and precaudal of Soleidae to analyse for species level.

Tables 4 and 5 show that the combined view allows for higher accuracies for almost all subset analyses, ranging from small increases to quite significant improvements.

**TABLE 4:** Average accuracy and standard deviation of bootstrapped (*n* = 100) classification to vertebra type and species for modern samples per view, considering all cervical, precaudal and caudal vertebrae.

Group	Anterior	Sinistral	Combined
Vertebra type	0.8202 ± 0.02	0.8281 ± 0.02	0.8953 ± 0.02
Family	0.6936 ± 0.73	0.7347 ± 0.02	0.8015 ± 0.02
Species	0.3803 ± 0.02	0.4082 ± 0.02	0.5729 ± 0.02

**TABLE 5:** Average accuracy and SD of bootstrapped (*n* = 100) classification to taxa for modern samples per view and vertebra type.

Vertebra type	Family level	Species level			
	Pleuronectiformes	Pleuronectidae	Plaice/flounder	Scophthalmidae	Soleidae
Atlas					
Anterior	0.8478 ± 0.07	0.4435 ± 0.13	0.774 ± 0.14	0.55 ± 0.24	—
Sinistral	0.7102 ± 0.08	0.4375 ± 0.11	0.74 ± 0.17	0.845 ± 0.22	—
Combined	0.8232 ± 0.07	0.4275 ± 0.15	0.794 ± 0.15	0.7475 ± 0.20	—
Cervical					
Anterior	0.7247 ± 0.06	0.5122 ± 0.07	0.704 ± 0.15	0.5636 ± 0.14	0.492 ± 0.18
Sinistral	0.7916 ± 0.05	0.5584 ± 0.07	0.7578 ± 0.12	0.4799 ± 0.20	0.804 ± 0.14
Combined	0.8441 ± 0.04	0.7752 ± 0.07	0.8129 ± 0.13	0.6065 ± 0.20	0.664 ± 0.15
Precaudal					
Anterior	0.9229 ± 0.03	0.6195 ± 0.06	0.7946 ± 0.08	0.6341 ± 0.11	—
Sinistral	0.8826 ± 0.04	0.6871 ± 0.05	0.8684 ± 0.06	0.9061 ± 0.07	—
Combined	0.9569 ± 0.02	0.8179 ± 0.05	0.9126 ± 0.05	0.9011 ± 0.07	—
Caudal					
Anterior	0.7538 ± 0.02	0.627 ± 0.04	0.7746 ± 0.06	0.8175 ± 0.07	0.8183 ± 0.08
Sinistral	0.7858 ± 0.03	0.652 ± 0.04	0.8535 ± 0.05	0.7268 ± 0.07	0.829 ± 0.07
Combined	0.8401 ± 0.02	0.7809 ± 0.03	0.9282 ± 0.04	0.8425 ± 0.07	0.9132 ± 0.06

The classification to vertebra type using all specimens is largely successful with an average accuracy of 89.5% to identify the specimen to the correct vertebra type using the combined view. For family level using all specimens, the accuracy is slightly lower (80.15%), while the accuracy is inadequate to use for species‐level identifications (57.29%). For the precaudal vertebrae, it is better to identify the specimens using a hierarchical system, with first classification to vertebra type and then family level (89.53% × 95.69% = 85.67%), as the probability of classifying the specimens correctly is higher than using a non‐hierarchical system (80.15%). For the cervical and caudal vertebrae, the probability of correctly classifying the family is higher when using a non‐hierarchical classification. When the vertebra type is known from visual inspection, it is best to directly classify it to family or species. For species identifications, the best result is obtained by first identifying to family and then to species.

Remarkably, there is a high classification accuracy for *P. platessa and P. flesus*, two species that are osteologically very similar. Using the atlas and cervical vertebrae, these two species can be distinguished from each other with ca. 80% (note large standard deviation, ca. 0.14) success rate, and using the precaudal and caudal vertebrae ca. 91% (small standard deviation, ca. 0.05). Classification of the modern dataset to right‐eyed or left‐eyed *P. flesus* was relatively accurate (53%–69% accuracy; see Table S3).

### 3.3 Identification of archaeological samples

Using the classification developed in the previous step, archaeological samples were attempted to be identified to family and species level. Table 6 summarises the identification success rate for each vertebra type per view, as verified by collagen peptide mass fingerprinting. Details of the analysis for the archaeological samples can be found in Tables S4–S10.

**TABLE 6:** Percentage of correctly identified archaeological samples to vertebra type, and to taxon using GMM when vertebra type is provided. Vertebra type verified by visual identification. Species verified by collagen peptide mass fingerprinting.

Species (ZooMS)	Species (GMM)		
	Anterior	Sinistral	Combined
Atlas vertebra			
Pleuronectidae (*n* = 8)	7 (87.5%)	7 (87.5%)	8 (100.00%)
*P. flesus* (*n* = 4)	1 (25.00%)	2 (50.00%)	2 (50.00%)
*P. platessa* (*n* = 4)	1 (25.00%)	2 (50.00%)	1 (25.00%)
Cervical vertebra (*n* = 11)	5 (45.45%)	5 (45.45%)	6 (54.55%)
Pleuronectidae (*n* = 11)	9 (81.82%)	9 (81.82%)	9 (81.82%)
*P. flesus* (*n* = 9)	0 (0.00%)	5 (55.55%)	2 (22.22%)
*P. platessa* (*n* = 2)	0 (0.00%)	1 (50.00%)	1 (50.00%)
Precaudal vertebra (*n* = 28)	16 (57.14%)	9 (32.14%)	15 (53.57%)
Pleuronectidae (*n* = 28)	26 (92.86%)	27 (96.43%)	25 (89.29%)
*P. flesus* (*n* = 16)	3 (18.75%)	9 (56.25%)	4 (25.00%)
*P. platessa* (*n* = 11)	2 (18.18%)	3 (27.27%)	4 (36.36%)
*L. limanda* (*n* = 1)	1 (100.00%)	0 (0.00%)	1 (100.00%)
Caudal vertebra (*n* = 58)	52 (89.66%)	54 (93.10%)	49 (84.48%)
Pleuronectidae (*n* = 58)	54 (93.10%)	55 (94.83%)	55 (94.83%)
*P. flesus* (*n* = 29)	8 (27.59%)	13 (44.83%)	13 (44.83%)
*P. platessa* (*n* = 27)	10 (37.04%)	7 (25.93%)	8 (29.63%)
*L. limanda* (*n* = 2)	1 (50.00%)	0 (0.00%)	1 (50.00%)

Compared to the modern dataset, the classification of vertebra type of the archaeological dataset is much less accurate (68%–73% vs. 82%–89%). A few vertebrae (two precaudal and two caudal vertebrae) could not be identified as these had too few landmarks remaining (only one or two), which were not sufficient to be run by the LDA. Even if these four samples would have been correctly identified, the success rate of the application on archaeological remains is lower compared to the accuracy obtained from the modern dataset.

When the vertebra type was able to be classified visually, the success rate for the family‐ and species‐level identifications were checked for the archaeological samples (Table 6). The success rate for family‐level identification is rather high for the archaeological samples and is not much lower than the average success rate for the modern dataset. For the caudal vertebrae, there is even a clearly higher success rate for the archaeological samples. Due to the absence of other families in the archaeological dataset, however, this success rate should be treated with caution. When looking at the success rate of the species identifications, there is a clear difference between the archaeological dataset and the modern dataset. In most cases, less than 50% of the archaeological samples are correctly identified to species. Furthermore, the analysis identified the samples to a variety of different species, which were mostly not recorded from the archaeological sites. Overall the sinistral view seems to be the most successful view to identify the archaeological material to species (40.00%). The combined view (35.24%) and the anterior view perform worse (26.67%).

As there is no way of verifying the sidedness of archaeological *P. flesus* samples confidently in this case study, the classification of right‐ and left‐eyed *P. flesus* is not further discussed. Several samples were classified as left‐eyed specimens for at least one view using this classification method per vertebra type and only one sample was classified as left‐eyed by all three views (Table S6).

## 4 DISCUSSION

### 4.1 Morphological variation between modern samples

Geometric morphometrics allows some distinction between vertebra type and taxa of modern flatfish vertebrae via analysis of 2D landmark configurations of two views. GMM on vertebra types is suitable for discrimination of vertebra types even if there is a large overlap between cervical, precaudal and caudal vertebrae. In the example we provided, the landmark configuration has been sub‐sampled to ensure the inclusion of anatomical landmarks shared within the sample.

Of the landmarks initially selected, some were discarded during the analysis, as it became clear they were either difficult to use consistently (see Table S11 and Figure S26) or were often missing in archaeological remains and contributed little to morphological variation, such as the arch tips (Figures S22–S24). Loss of the arch tips landmarks reduces the morphological variation caused by the position of the vertebra along the spinal column.

Shape differences between vertebra types showed better performance in anterior view, as vertebra types differ strongly in the implantation of the haemal arches, which shows best in the anterior view. One of the easiest ways to visually distinguish between vertebrae of different Pleuronectiformes taxa is the general shape of the vertebra, but also the surface of the lateral side of the centre of the vertebra, which often consists of several ridges running anteroposterior along the centre. These ridges, however, could not be landmarked as there do not seem to be any clear homologous structures present between the taxa, losing potentially very diagnostic features for the GMM analysis and resulting in limited separation in the PCA between the vertebra types.

From the analysis on the modern reference samples, the anterior and combined views are best to distinguish between taxa using the atlas vertebrae. The cervical vertebrae cannot be used to distinguish taxa using PCA. This is partly due to the reduced number of landmarks available, as the haemal processus are absent, but could also be due to the limited morphological variation between taxa. Precaudal and caudal vertebrae can be used in several cases to distinguish between taxa, although in most cases the separation is not clear and there remains some overlap between the taxa on the principal component plot.

The atlas vertebra seems to allow for much better distinction between taxa than the other vertebra types, which could be due to the more distinct shape of the centre and the presence of the condyles. These articulate with the neurocranium, which provides more taxon‐specific morphological shape variation (e.g. Wouters et al., 2007).

Cervical vertebrae are in the transition zone from the neurocranium to the body. Therefore, the size and shape of structures such as the onset of the haemal arch and the implantation of the neural arch, differ strongly between these few vertebrae at the beginning of the spinal column. This could create a greater morphological difference between vertebrae from the same individual than between vertebrae from different species.

The precaudal vertebrae in general seems to show the best distinction for all taxa and all views, but also here a slight effect of the changes along the spinal column could create noise in the analysis. Along the caudal vertebra series, PCA discriminates Soleidae from the other families.

In this dataset it seems that precaudal vertebrae—and potentially atlas and cervical, although more samples are needed to verify this—do show a slight morphological distinction between right‐ and left‐eyed *P. flesus* as seen in the PCA using the anterior view, where the asymmetry can be best detected. By applying GMM on the archaeological record, this could potentially reveal the presence of many reversed flounder in assemblages, as there are not many reversed flounder bones reported so far from archaeological sites. Distinguishing between these two forms could reveal more about the populations and ecology of exploited flounder, as the abundance of reversed flounder is geographically dependent and could impact the ecology of the individual fish (Fornbacke et al., 2002; Russo et al., 2012).

Although the distinction between taxa is limited, there does seem to be slight differences in shape between vertebra types and taxa in specific subsets, meaning it could potentially be possible to identify vertebrae by comparing their shape with this modern reference dataset.

### 4.2 Classification of modern samples

A high mean accuracy was obtained using the bootstrap classification test on modern specimens showing the potential to use GMM to identify species of flatfish using vertebrae.

From the LDA the approach in combining views shows higher classification accuracies compared to the anterior and sinistral views individually. It is therefore recommended to use the combined view approach for identification analyses. The improved accuracies can easily be explained by the increased amount of morphological information present in the dataset when combining different views together. This approach can be used to simulate a 3D methodology and can be of use when 3D modelling is not possible due to time constraints, issues with accessibility to scanning material or analysing software, or when two separate landmark datasets are required when dealing with non‐spatially linked objects or living organisms. When available, however, a 3D approach is preferable (Profico et al., 2019). Only for atlas vertebrae does the anterior seem to be better at classifying families and Pleuronectidae than the combined view. This might be due to the presence of the condyles in the anterior view, providing crucial diagnostic shape information, without the noise originating from the sinistral view. Precaudal and caudal vertebrae have a higher mean accuracy for species‐level classification than atlas and cervical vertebrae, which could be related to the larger sample size for these subsets.

The mean accuracies are rather high for most subsets, indicating that the little morphological distinction between taxa is enough for the analysis to work in most cases, albeit not perfect. The high accuracy for *P. platessa* and *P. flesus* is remarkable, which shows that there is ample morphological distinction between these two species. This contrasts with the lack of clear diagnostic features found between these two species so far using conventional visual morphological identification (Wouters et al., 2007). The lowest mean accuracies were consistently noticed for Pleuronectidae. The mean accuracy to distinguish between species in this family was even lower than 0.5 for the atlas vertebrae. This family contains many species with similar morphology of the vertebrae. The lower accuracy could be due to the lack of distinction between the taxa and potentially also due to an inadequate number of specimens per species included in this study compared to the number of species in this family.

Contrary to what was expected is the low accuracy of determining the sidedness of *P. flesus* for cervical and precaudal samples. This can, however, be explained by the small sample size of these two subsets. For the caudal vertebra, the success rate is rather high with a mean accuracy of 0.69 for the combined view and 0.68 for the anterior view. As there is some distinction between both forms on the PCA plot (see Figure S25) for the precaudal vertebrae, it can be expected that the accuracy can be increased if more samples are available to use as reference material to allow for a comparative classification.

This classification method could be used in the future to look for any diagnostic features that would allow visual identification of flatfish vertebrae, as mentioned above for *P. flesus* and *P. platessa*. It would be possible to assess which landmarks contribute the most to the differentiation of taxa. Potentially, these could be used to describe a visual identification method as well. Furthermore, assessing which landmarks are required for successful and accurate classification could help to select vertebrae for analysis that minimally have these essential landmarks present, which can avoid unnecessary analyses.

### 4.3 Identification of archaeological samples

**FIGURE 6:**
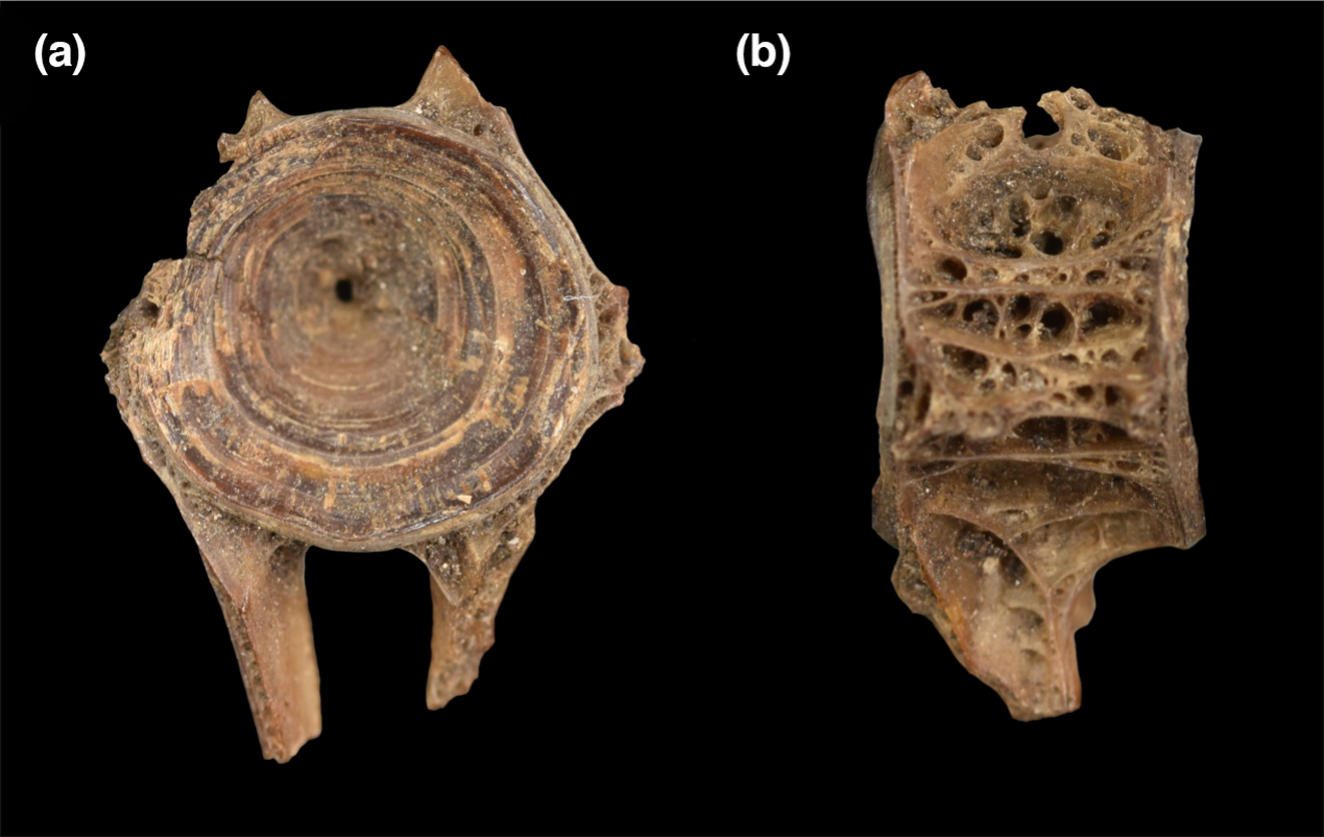
Example of an archaeological sample analysed in this study (COP0339), showing the preservation and lack of landmarks. (a). anterior view; (b). sinistral view.

The identification success of vertebra type, family level and species level on archaeological samples is lower than for the modern dataset as expected due to the generally poor preservation of archaeological remains which reduces the number of available landmarks for analysis (Figure 6, Table S9). This results in a lower accuracy of the classification system, meaning only well‐preserved samples might be able to be identified using GMM. In four cases, the few numbers of landmarks present, even hindered the analysis, as the analysis could not be run on samples with only two or fewer landmarks present. As was noticed during the analysis, however, even archaeological samples that were not severely fragmented, occasionally had low identification success. In addition to fragmentation, preserved archaeological bones can also become deformed during taphonomic processes, which may alter the shape of bones. No sample analysed in this study showed clear deformation visually and it is therefore thought that this has only minimally affected this analysis.

The type of a vertebra is correctly identified in around 70%–75% of cases using GMM. As can be expected, the anterior view works best overall to classify the vertebra type using GMM, as this view allows the best interpretation of the presence and shape of haemal arches. The sinistral view seems to work well with caudal vertebrae, which can be explained by the stronger inclination of the base of the arches in these vertebrae, which can be detected by GMM. Even with the bases of the haemal arches present, GMM still classifies some vertebrae incorrectly, making GMM potentially less reliable than a visual classification to vertebra type.

Family‐level identifications are relatively successful on archaeological material compared to modern specimens, while species‐level identifications are much less accurate. As fragmentation usually occurs on the arches and spina, it could be that these structures are more important for species‐level identifications than for the family‐level identifications. Furthermore, identification is somewhat difficult due the small shape variation observed between species, which is more apparent within a family than between families. This is especially a limiting factor for Pleuronectidae which affects the applicability of the method. When only considering *P. platessa* and *P. flesus* in the dataset, there are clear improvements for the species identification compared to the situation where all species are included in the analysis (see Table S7), although the species identifications are less accurate than in the modern dataset. Contrary to what was found during the classification test on modern material, the sinistral view has the highest success rate on archaeological material to identify a sample to the correct species. It may be that the fragmentation of landmarks in the anterior view has a larger effect on the success rate than the fragmentation of landmarks in the sinistral view.

As no other landmarks could be defined other than the ones used and trialled here, alternative shape analysis approaches could potentially be applied to see if these have a higher success rate of identifying archaeological samples. One possible approach is the addition of semi‐sliding landmarks, which can capture the curvature of the centrum of the vertebrae in anterior view, as has been done by Guillaud et al. (2016). This would, however, only be of use for well‐preserved archaeological specimens and modern specimens with a complete centrum. In this case study, semi‐sliding landmarks were not used as many of the used archaeological specimens showed fragmentation of at least part of the centre, making these types of landmarks difficult to use. Also, 3D approaches (e.g. Caro et al., 2019; Gabelaia et al., 2018; Sztencel‐Jabłonka et al., 2009) and machine learning algorithms, such as neural networks (e.g. Rauf et al., 2019; Storbeck & Daan, 2001), are other alternative approaches, of which the latter can be used potentially to include some of the more nuanced and non‐landmarkable features in the analysis, such as the ridges running along the lateral side of the vertebrae, and to improve the predictive power of classification approaches (e.g. Courtenay et al., 2019).

The combination of a limited shape variation and fragmentation of the archaeological samples is the most likely reason why geometric morphometrics is not a reliable tool for species identification of archaeological remains of flatfish, as fewer than 50% of the samples are correctly identified at species levels. On the contrary, at family level the correct identification is successful about 87.5%–96.4% of the time. Alternative identification methods, such as collagen peptide mass fingerprinting (e.g. Dierickx et al., 2022) and DNA (e.g. Kijewska et al., 2009; Pappalardo & Ferrito, 2015), are still recommended to differentiate between vertebrae of different species of archaeological flatfish in the North Sea area.

## 5 CONCLUSION

Landmark‐based geometric morphometric analysis on modern samples shows good performances in discriminating vertebra types and taxonomic status at family level. The application of the proposed protocol on 105 flatfish vertebrae from the North Sea revealed that this technique is unreliable to identify archaeological material of Pleuronectiformes to species as fewer than 50% of analysed samples are correctly identified. This is most likely due to the combination of a lack of morphological shape variation between taxonomic groups and the fragmentation of archaeological material. The provided datasets (modern and archaeological samples) and methodology could be a valid resource to researchers dealing with the challenging task of identifying vertebra type and taxonomic status of fish vertebrae from the archaeological record.

## AUTHOR CONTRIBUTIONS

Katrien Dierickx and Antonio Profico were involved in conceptualisation, methodology, software, formal analysis, investigation, visualisation, writing—original draft and writing—review and editing. Tarek Oueslati was involved in resources and writing—review and editing.

## CONFLICT OF INTEREST STATEMENT

We declare no conflict of interest in relation to this study.

## Supporting information


Figure S1.

Data S1.

Rscript ‐ GMM Pleuronectiformes ID.

## Data Availability

The script of the analysis is provided in the SI. Photos and TPS files of all specimens can be found on Zenodo following this doi: 10.5281/zenodo.7581135
