# Supplementary material for: Geometric morphometric analysis of Pleuronectiformes vertebrae: A new tool to identify archaeological fish remains?
Source: J Anat. 2023 Jul 26;243(6):982–96. doi: 10.1111/joa.13934 (PMC10641040; doi:10.1111/joa.13934)
Supplement: Supplementary file 1 — Figure S1. [file JOA-243-982-s003.docx]

# Geometric morphometric analysis of Pleuronectiformes vertebrae: a new tool to identify archaeological fish remains?

**Authors**: Katrien Dierickx^1,2^, Tarek Oueslati^3^, Antonio Profico^4^

**Affilitations**:

^1^Department of Archaeology, University of York, Heslington, York, UK

^2^Department of Archaeology and Cultural History, NTNU University Museum, Trondheim, Norway

^3^Centre National de la Recherche Scientifique, University of Lille, Lille, France

^4^Department of Biology, University of Pisa, Pisa, Italy

^1^Contacting author: [katrien.dierickx.icht@gmail.com](mailto:katrien.dierickx.icht@gmail.com)

ORCID: KD 0000-0002-9028-7652; TO 0000-0002-2886-085X; AP 0000-0003-2884-7118


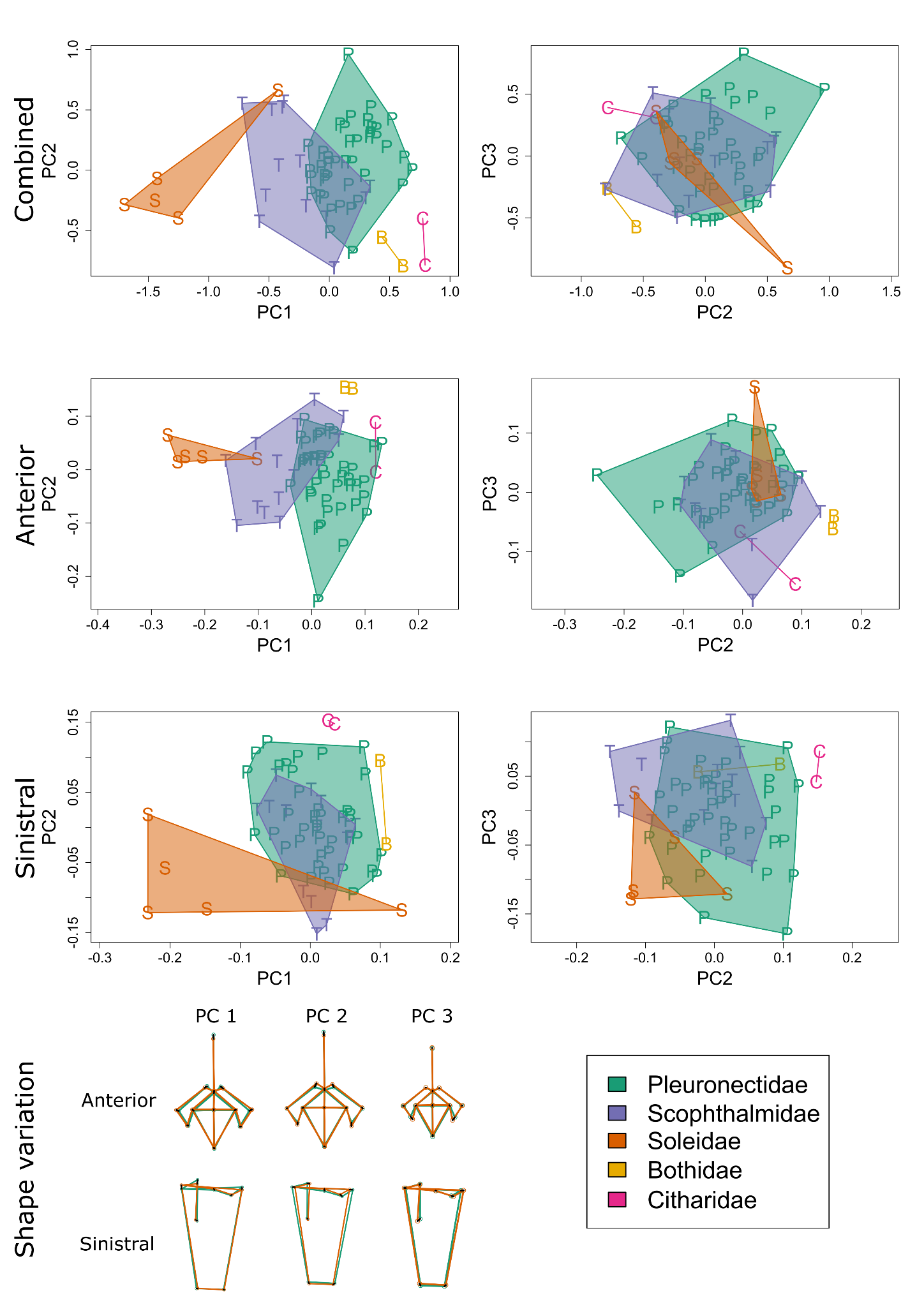

Figure S1. PCA and deformation grids of the atlas vertebra per family for the different views.


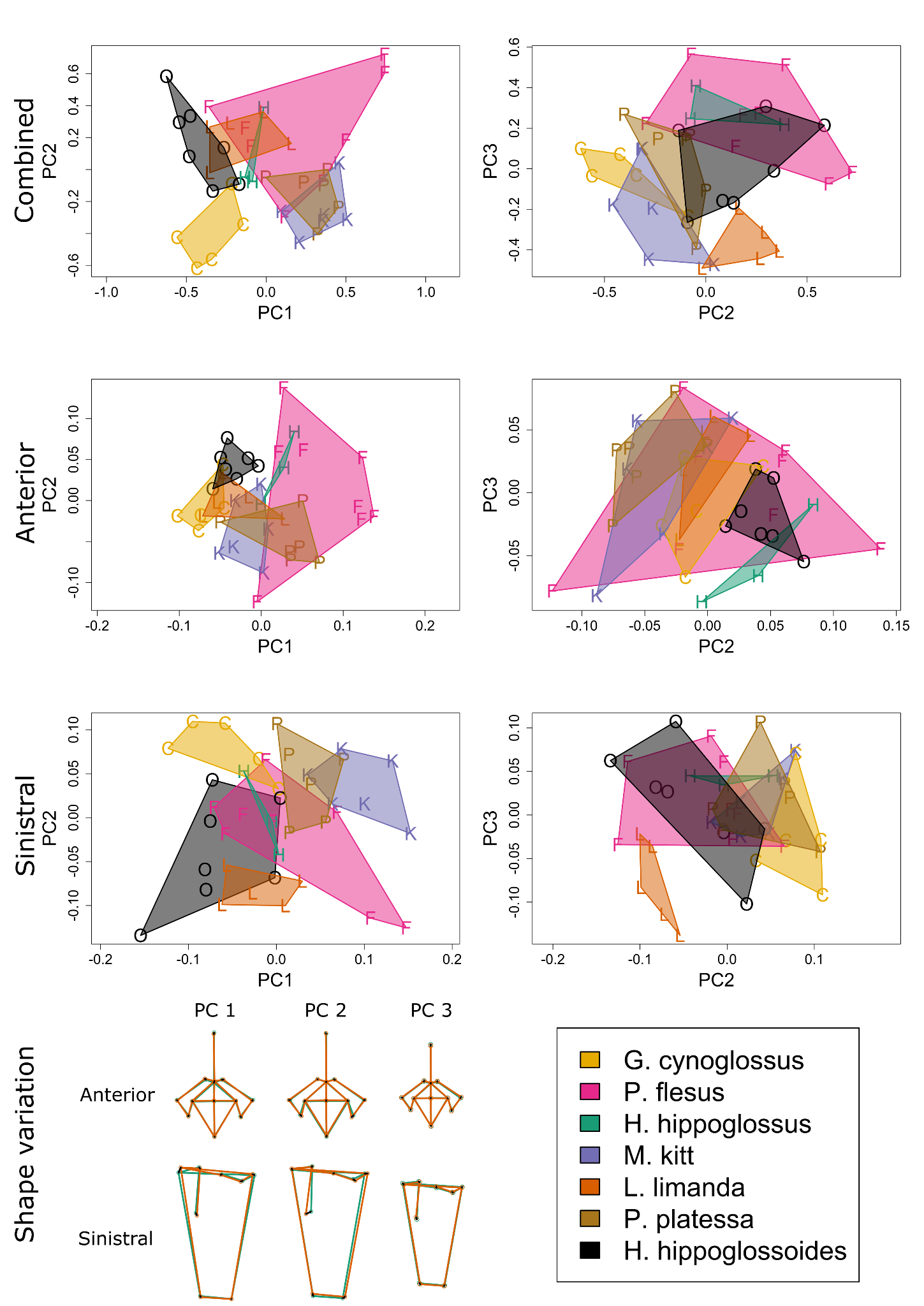


Figure S2. PCA and deformation grids of the atlas vertebra of Pleuronectidae for the different views.


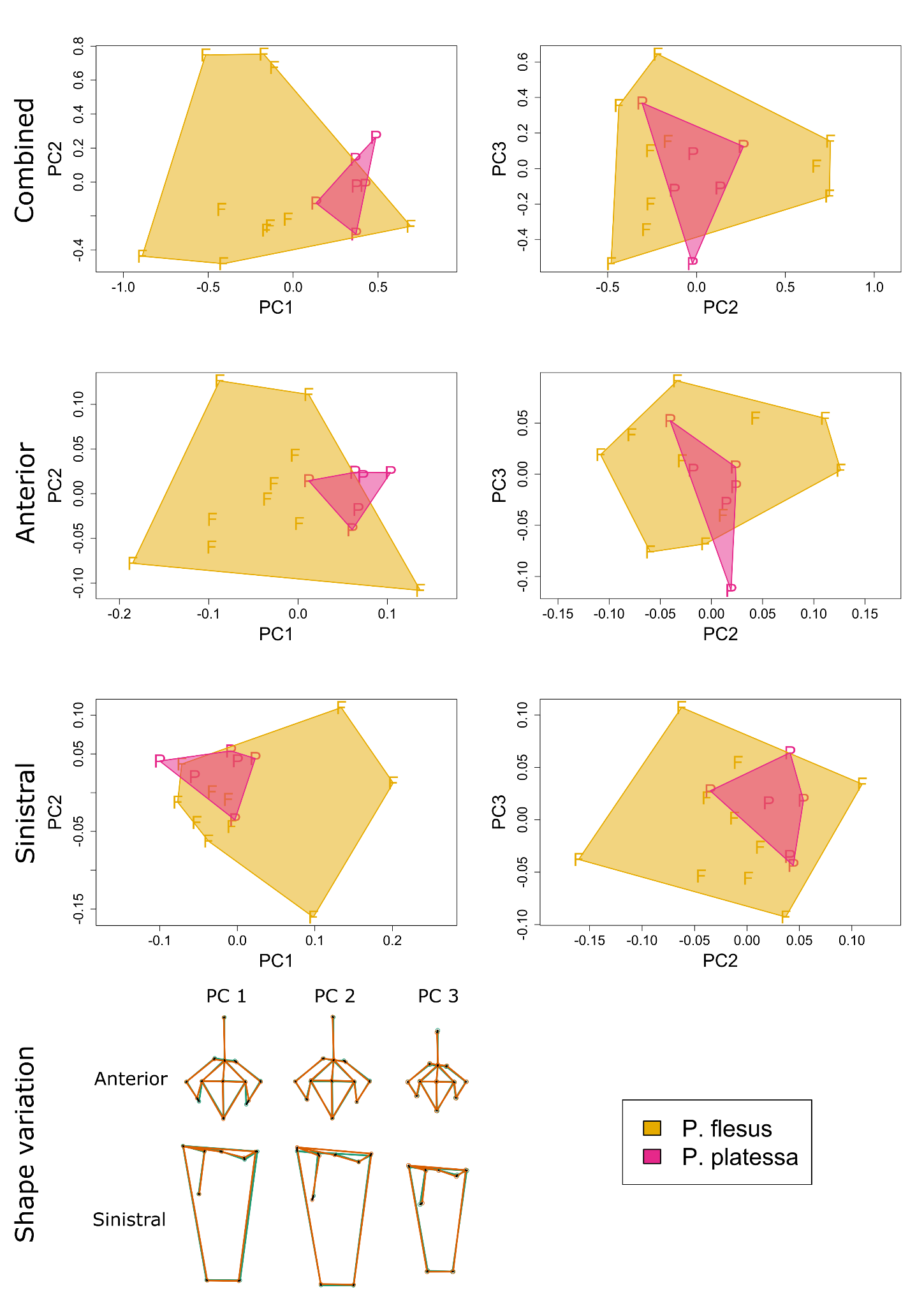


Figure S3. PCA and deformation grids of the atlas vertebra of plaice and flounder for the different views.


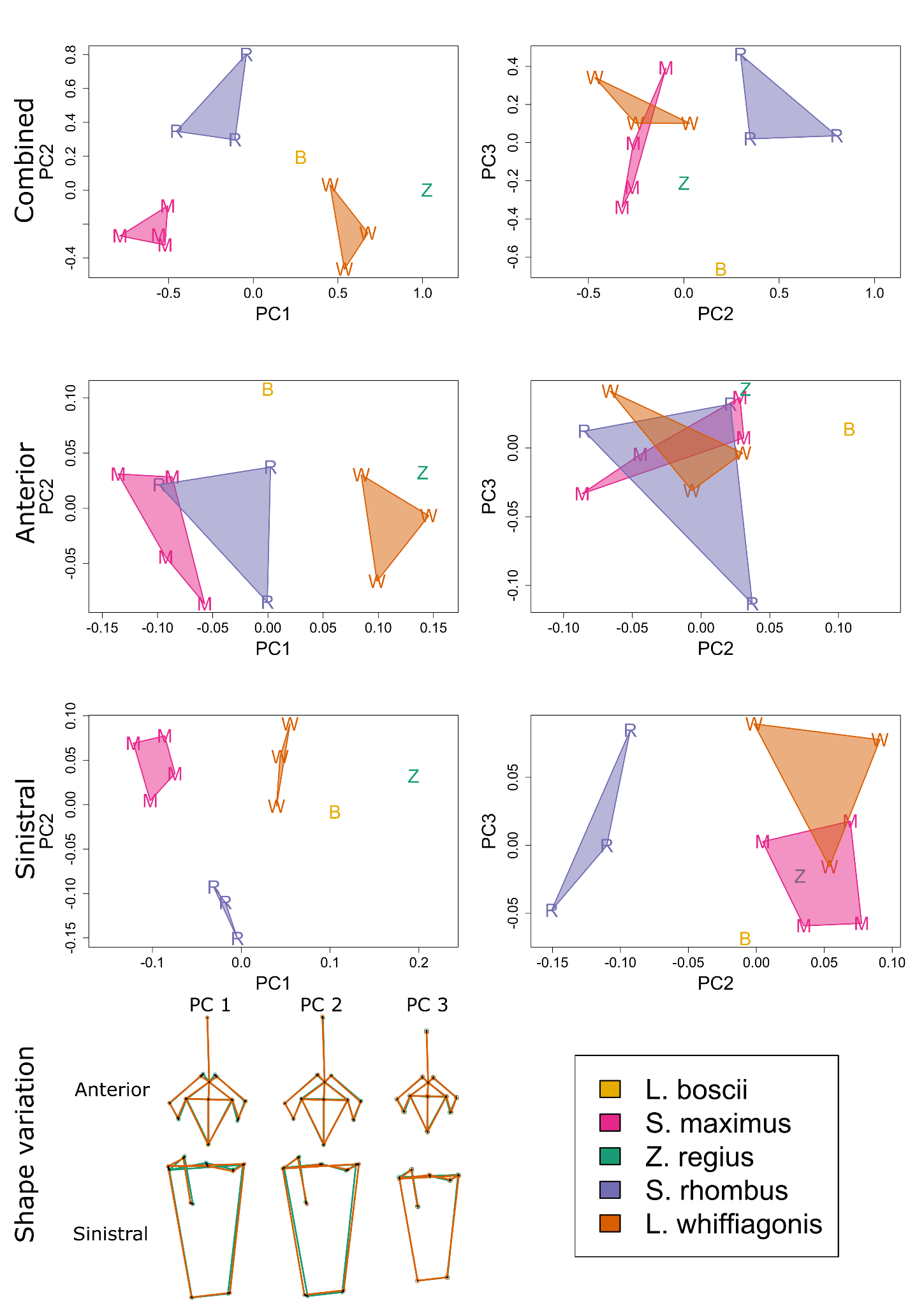


Figure S4. PCA and deformation grids of the atlas vertebra of Scophthalmidae for the different views.


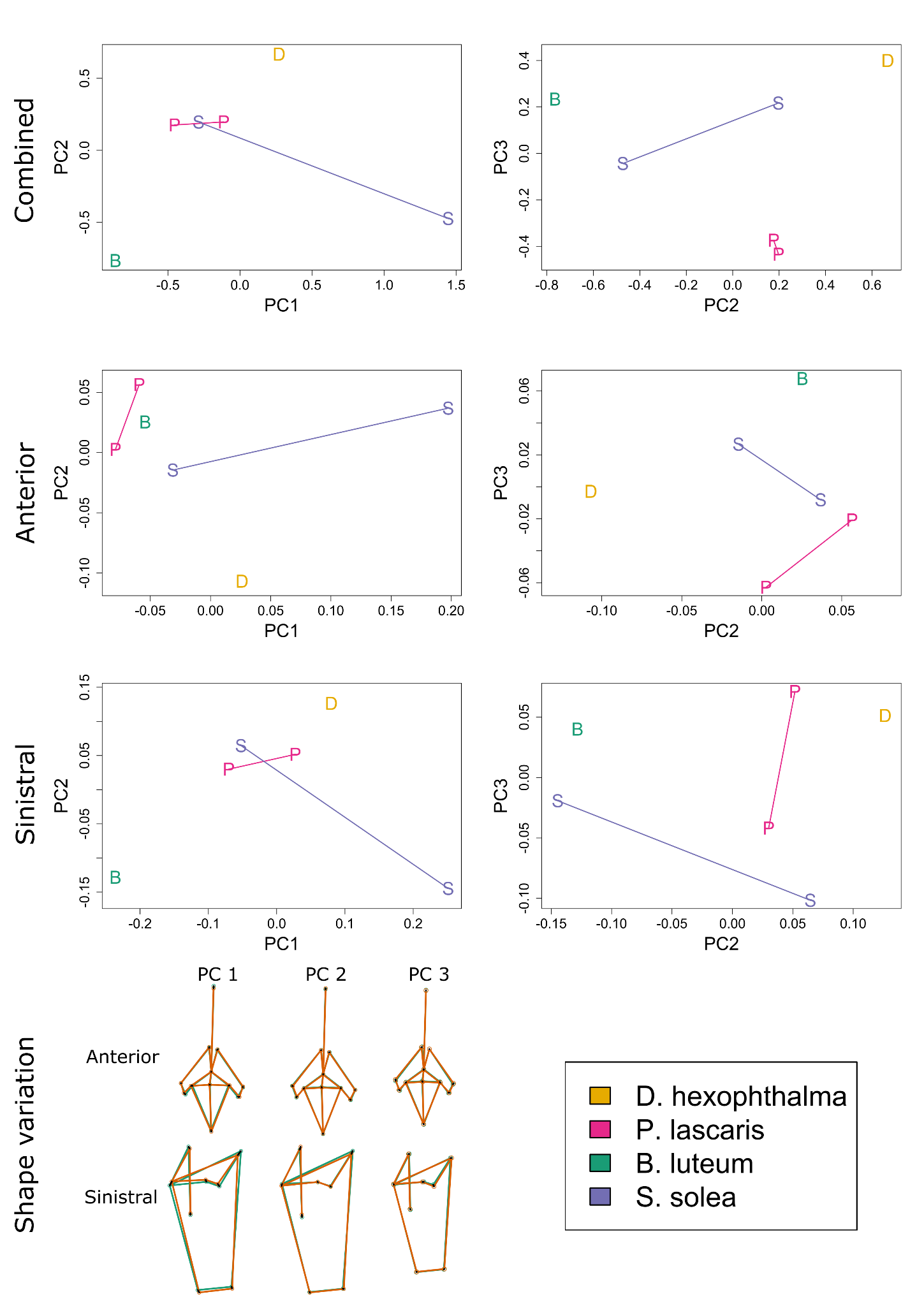


Figure S5. PCA and deformation grids of the atlas vertebra of Soleidae for the different views.


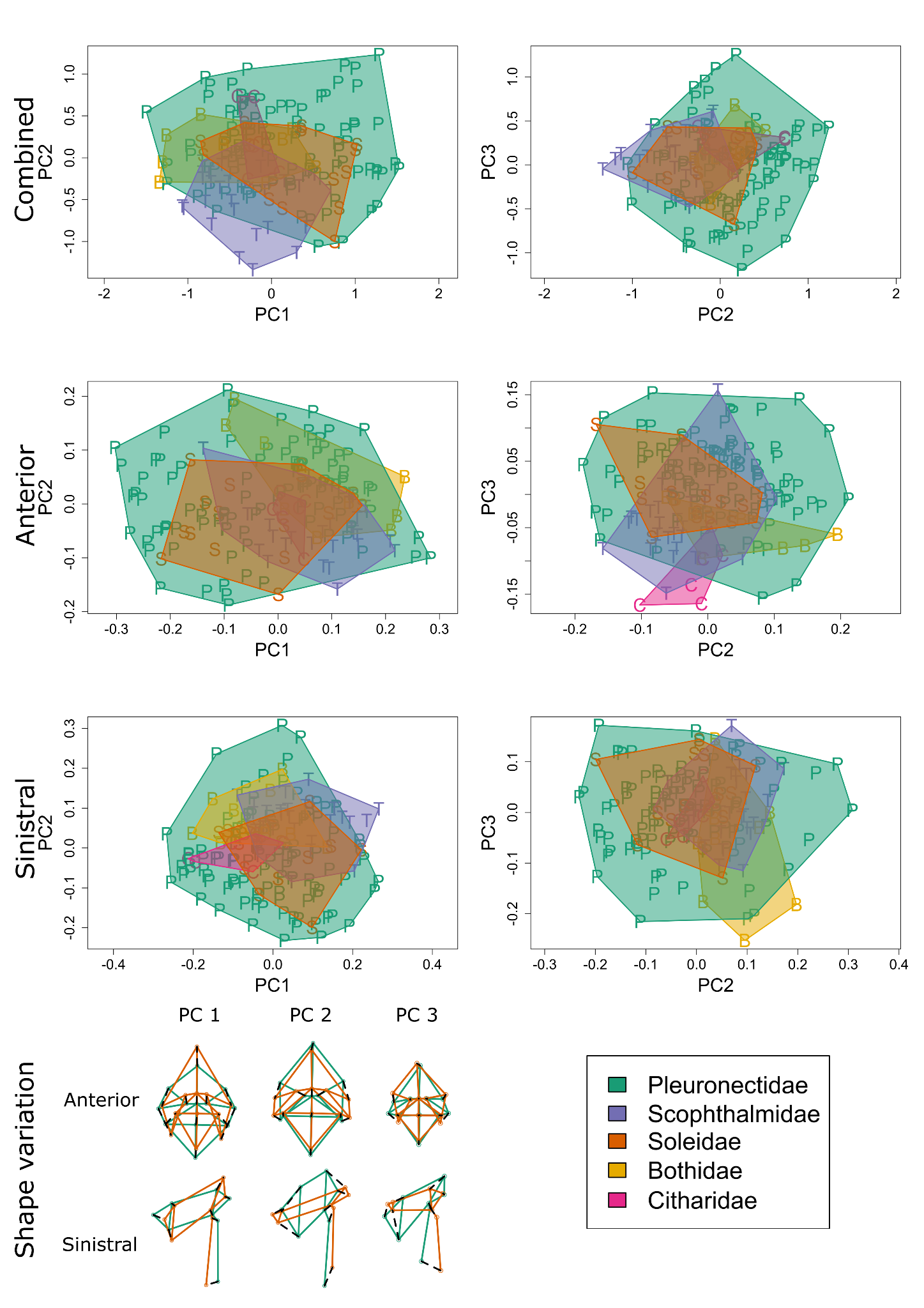


Figure S6. PCA and deformation grids of the cervical vertebra per family for the different views.


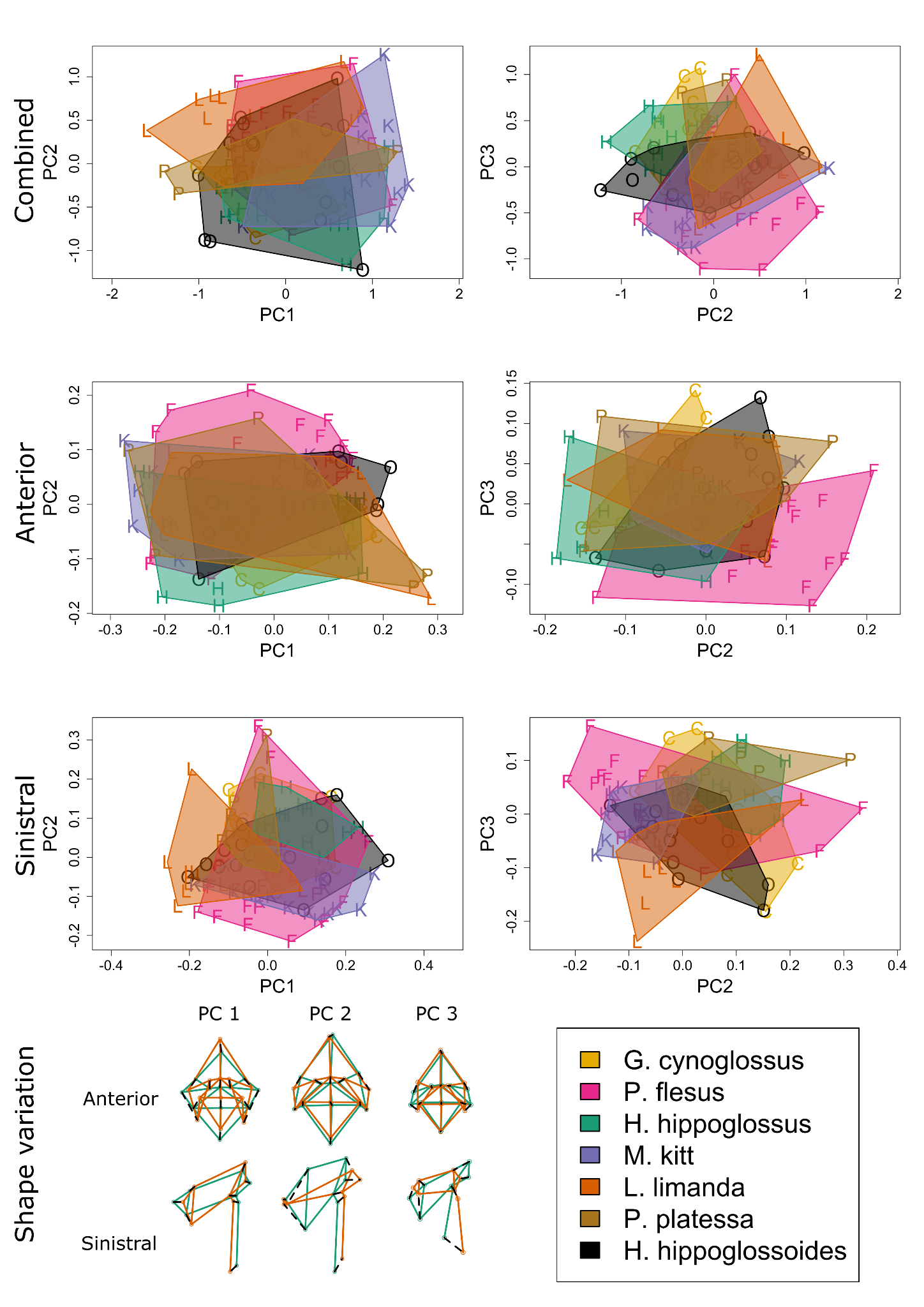


Figure S7. PCA and deformation grids of the cervical vertebra of Pleuronectidae for the different views.


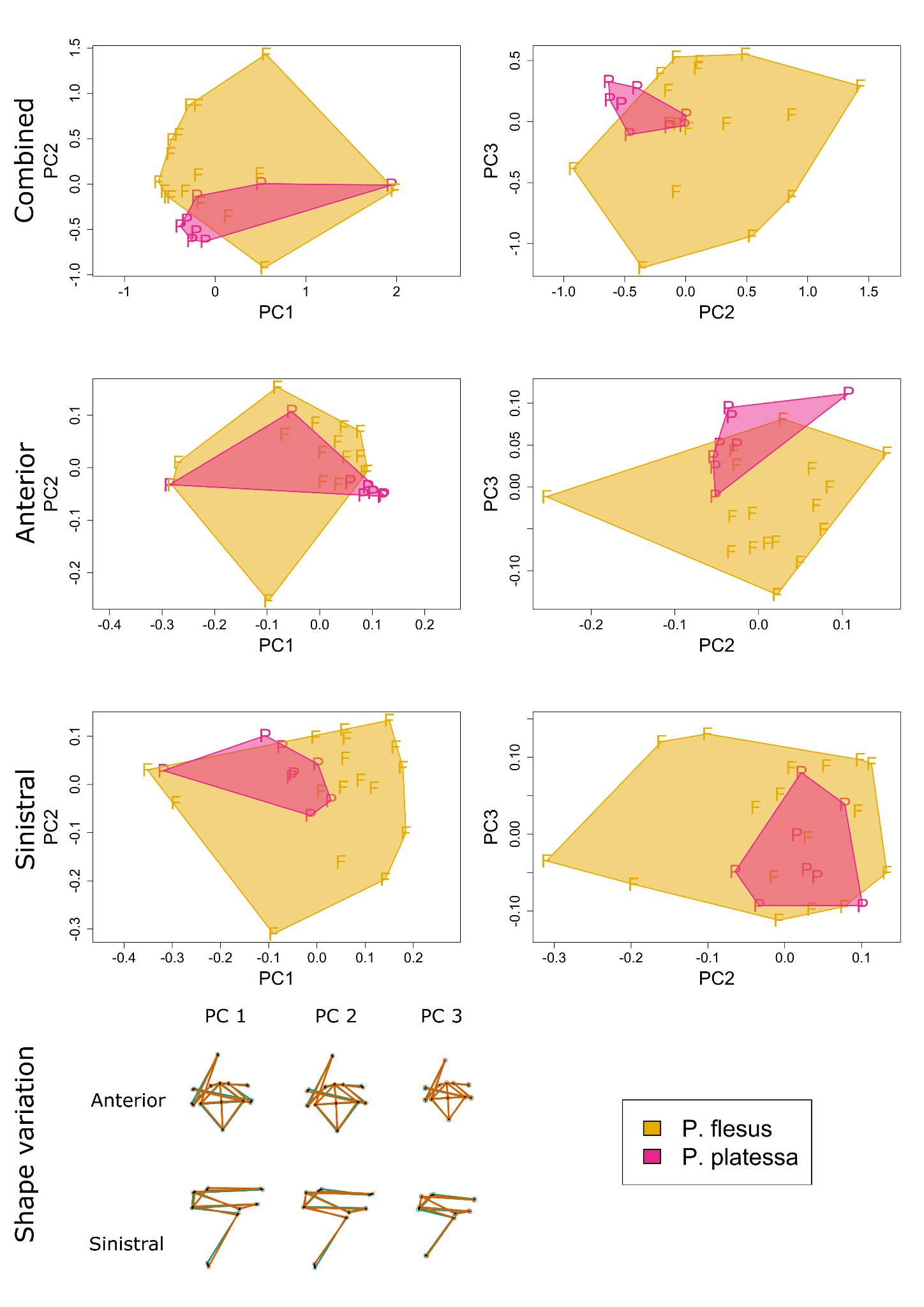


Figure S8. PCA and deformation grids of the cervical vertebra of plaice and flounder for the different views.


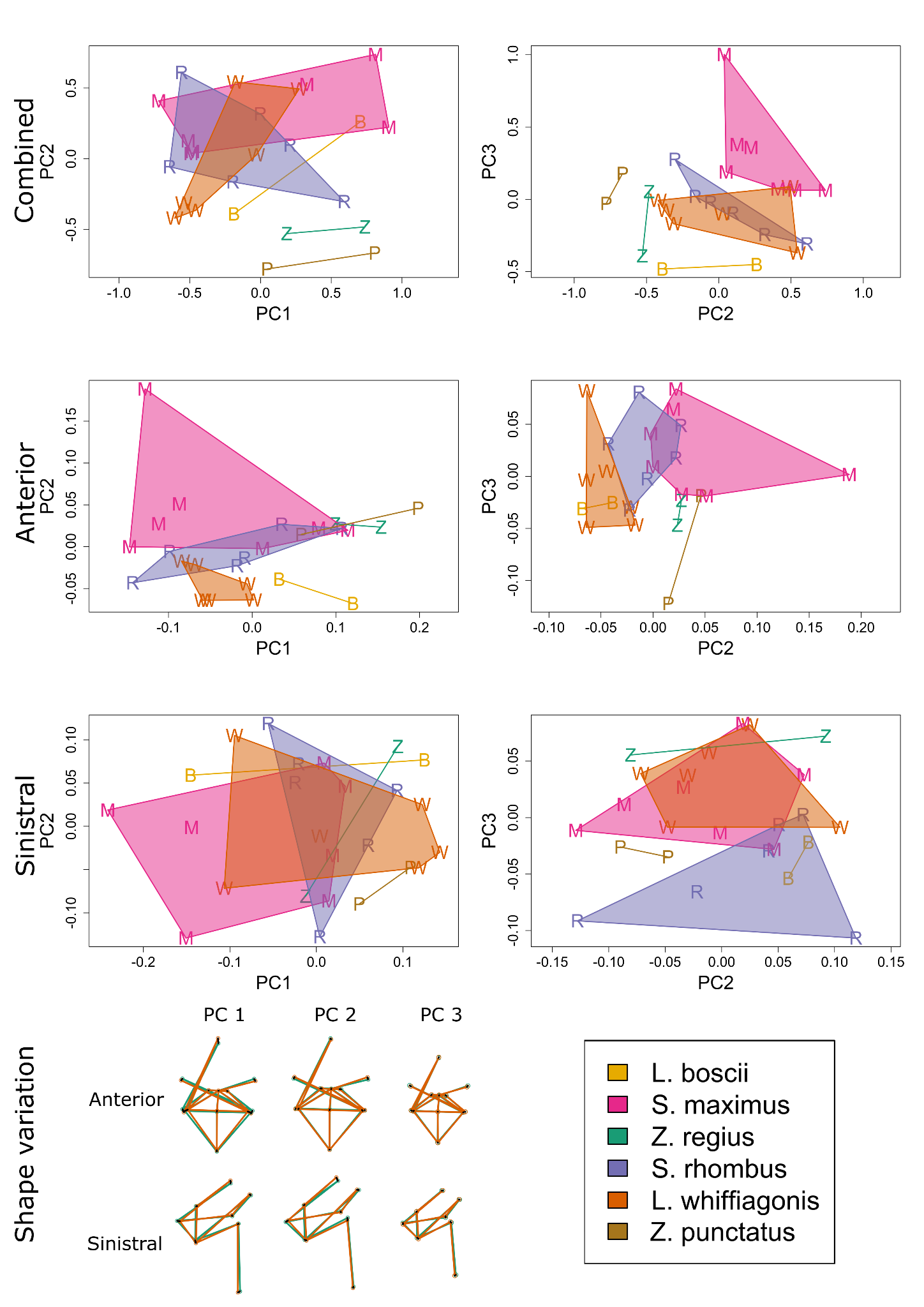


Figure S9. PCA and deformation grids of the cervical vertebra of Scophthalmidae for the different views.


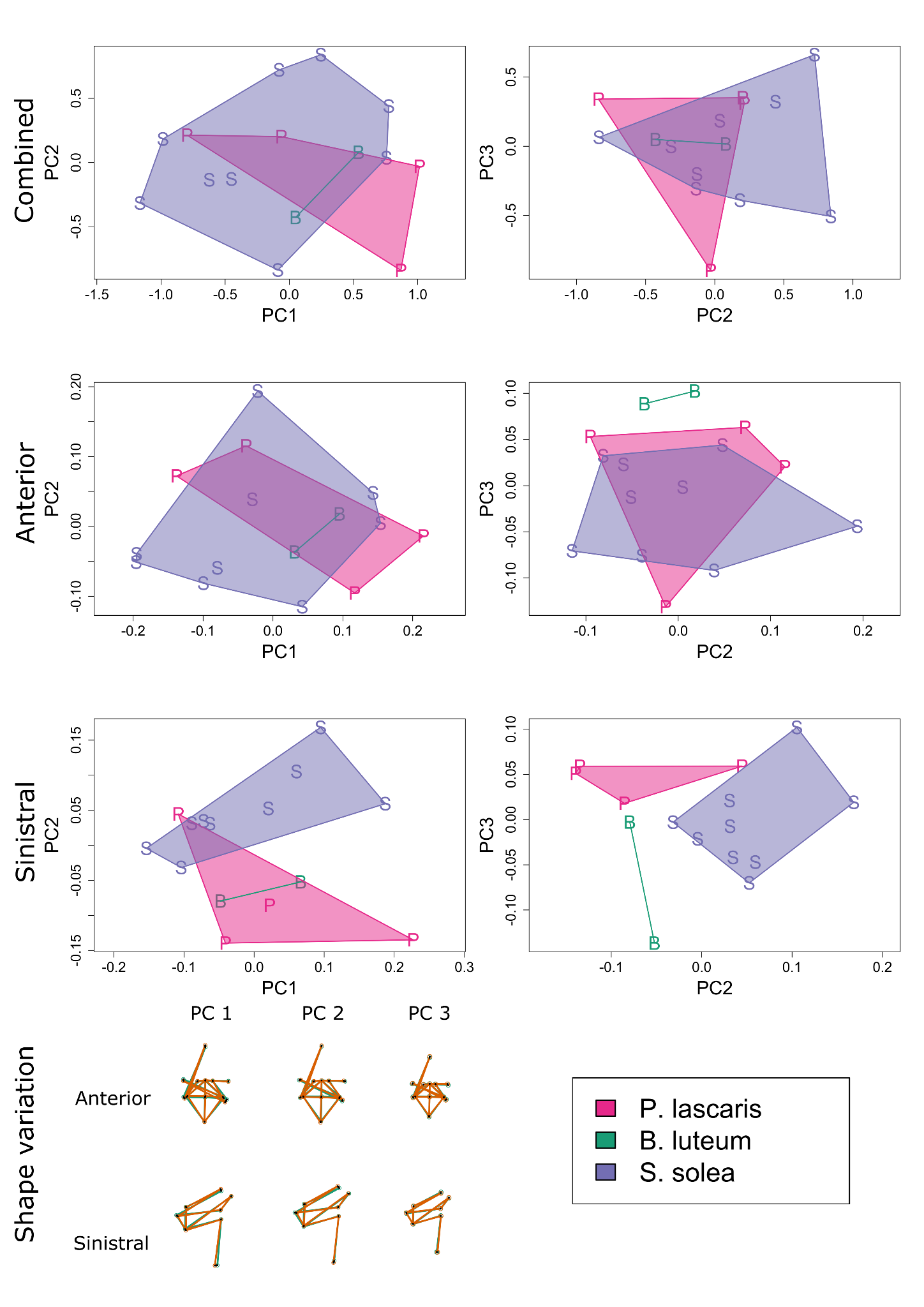


Figure S10. PCA and deformation grids of the cervical vertebra of Soleide for the different views.


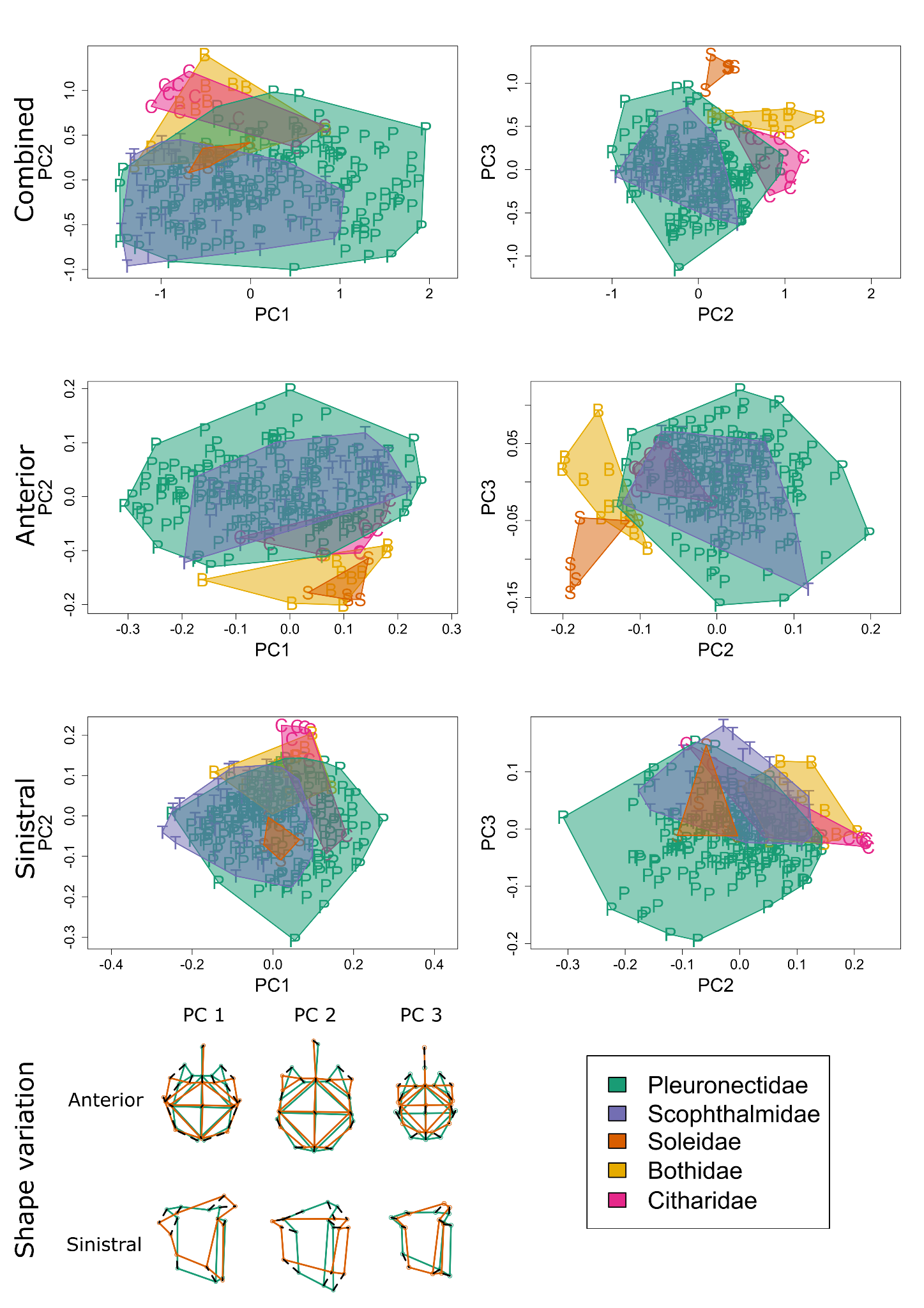


Figure S11. PCA and deformation grids of the precaudal vertebra per family for the different views.


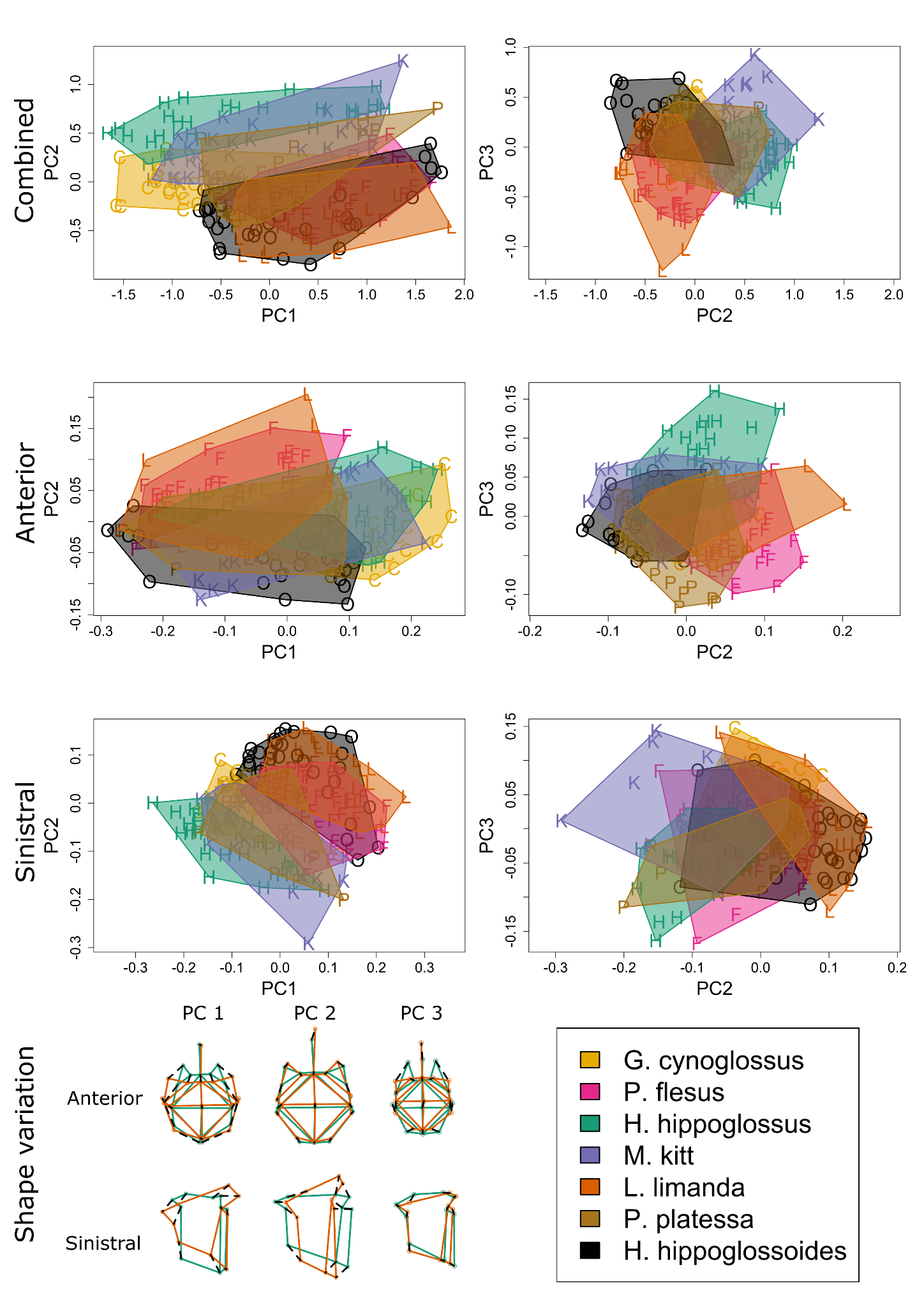


Figure S12. PCA and deformation grids of the precaudal vertebra of Pleuronectidae for the different views.


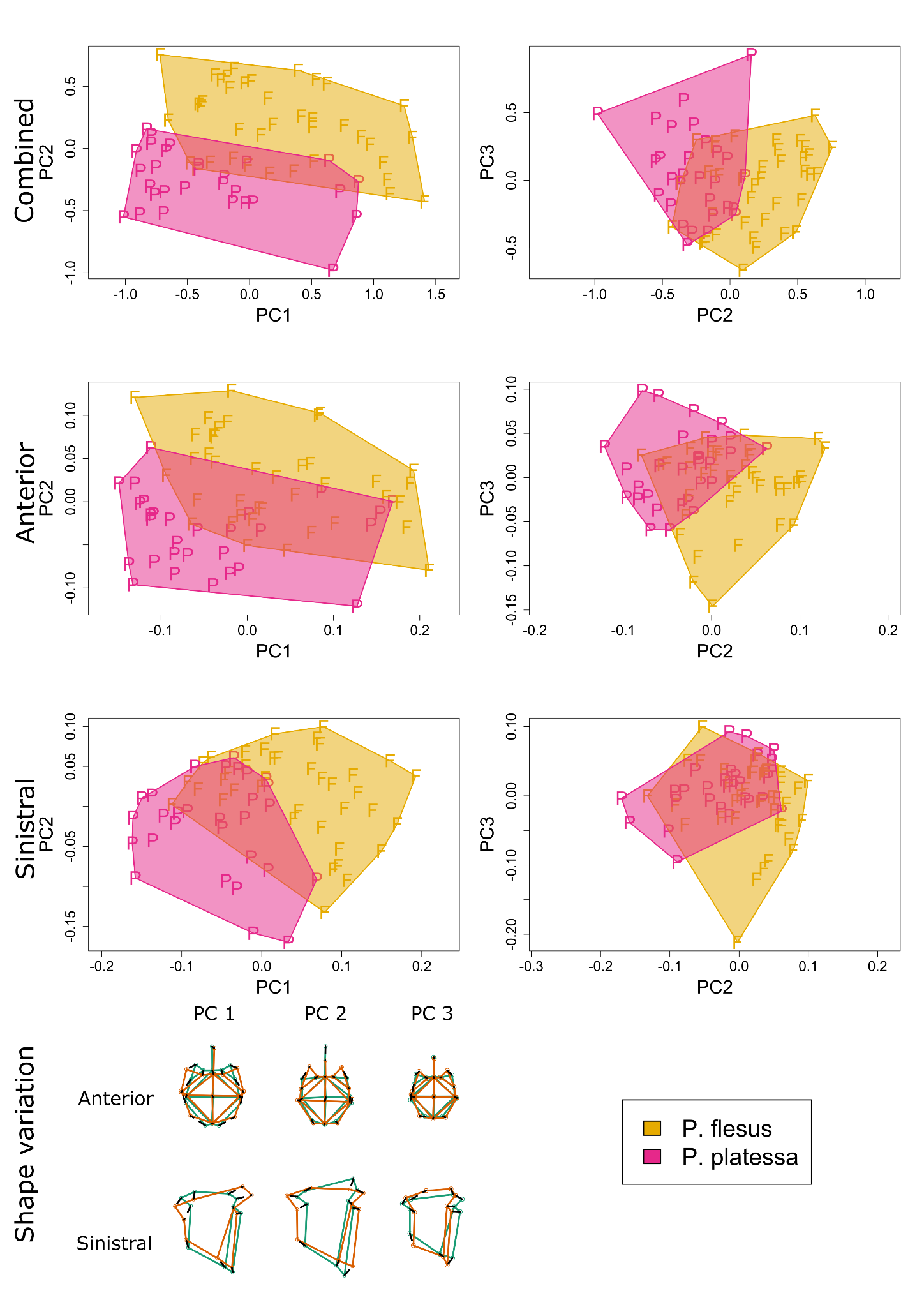


Figure S13. PCA and deformation grids of the precaudal vertebra of plaice and flounder for the different views.


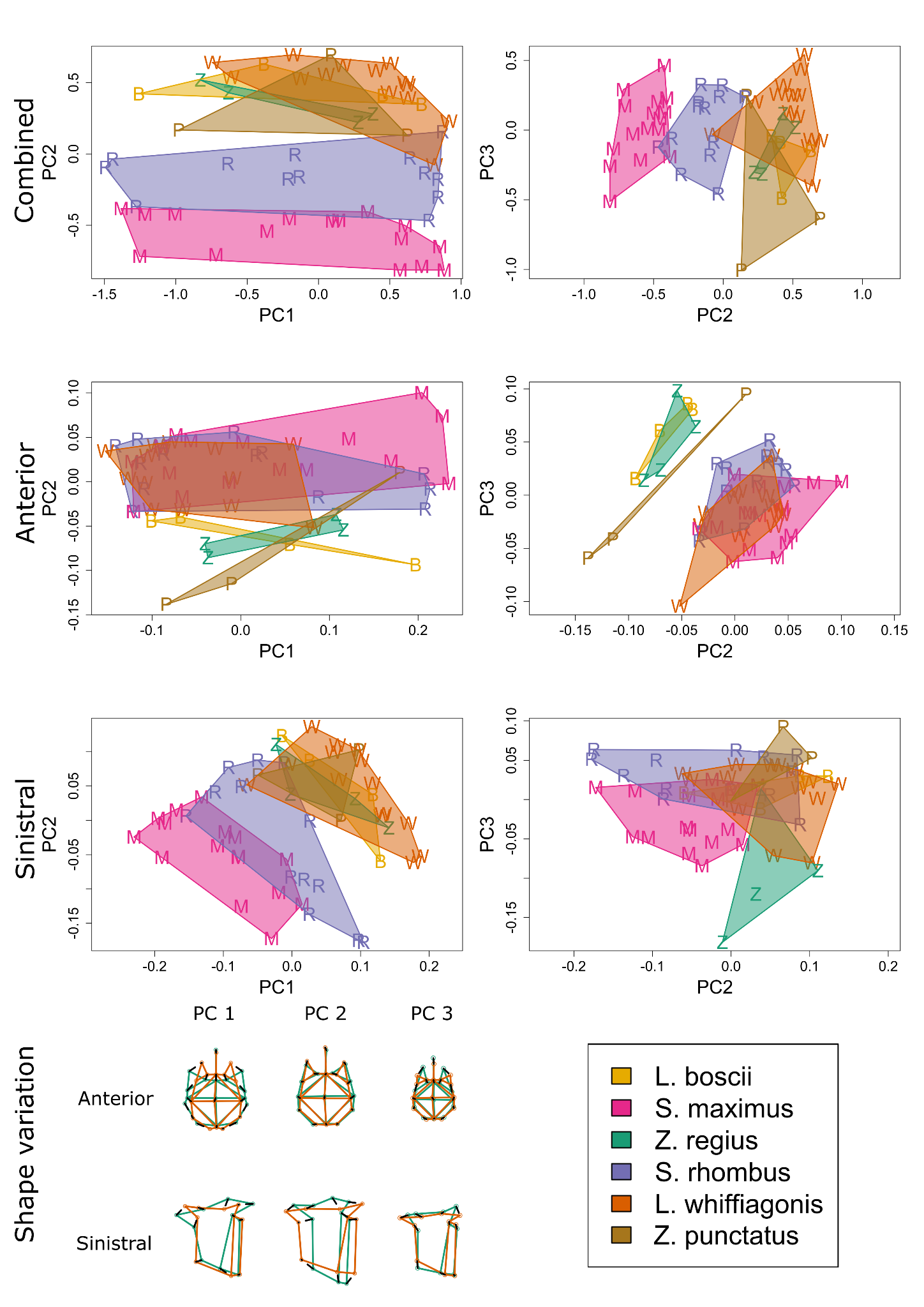


Figure S14. PCA and deformation grids of the precaudal vertebra of Scophthalmidae for the different views.


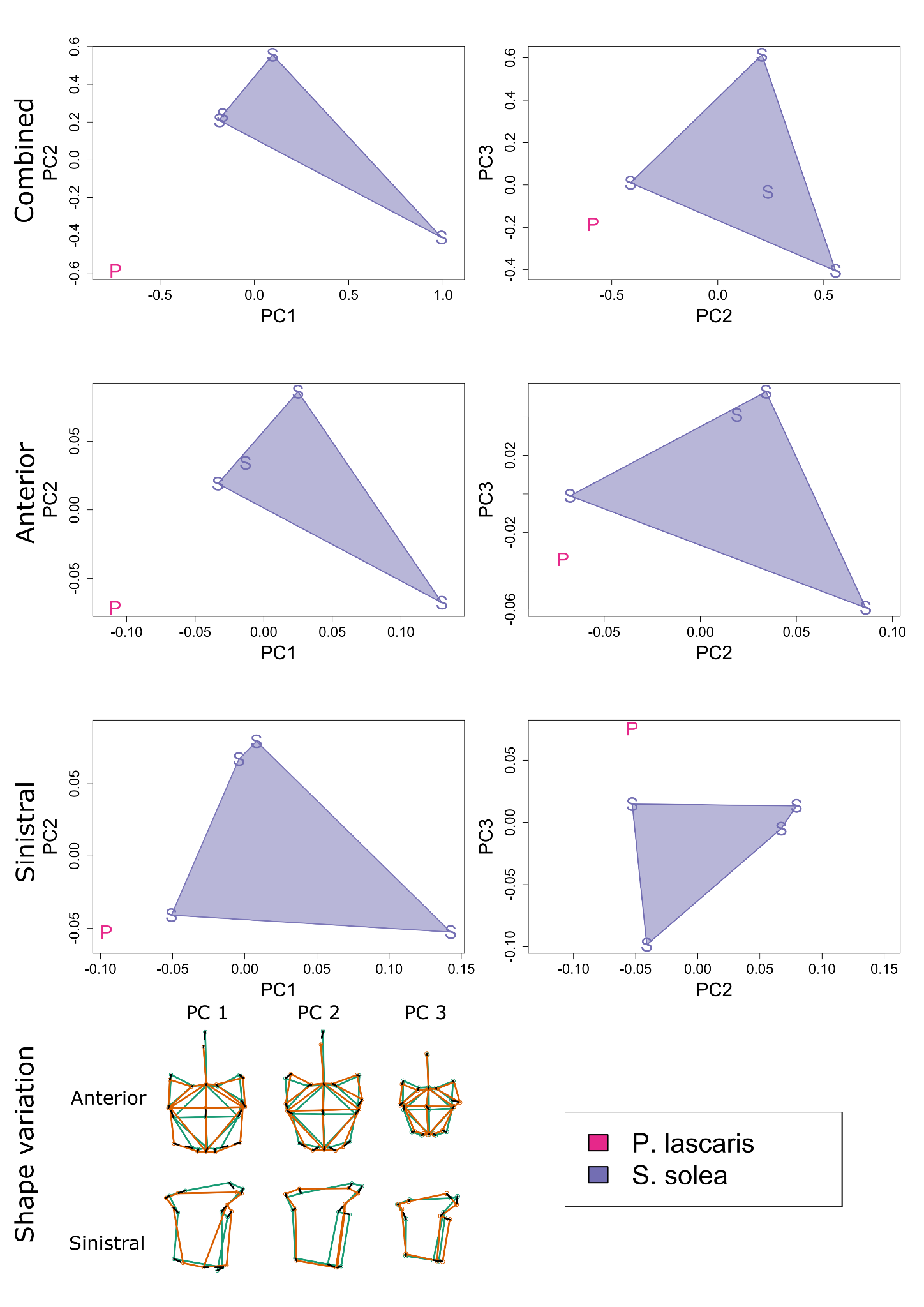


Figure S15. PCA and deformation grids of the precaudal vertebra of Soleidae for the different views.


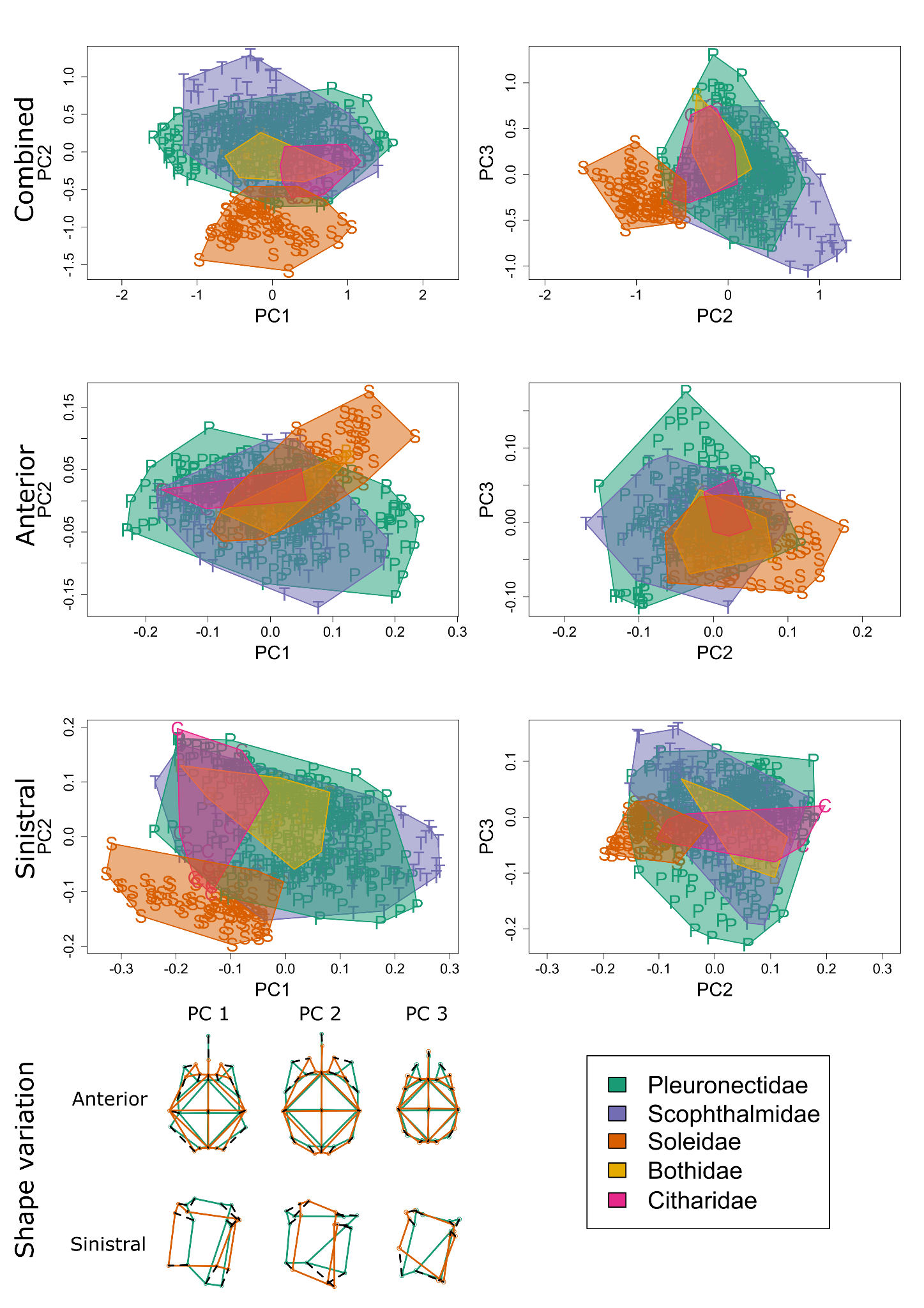


Figure S16. PCA and deformation grids of the caudal vertebra per family for the different views.


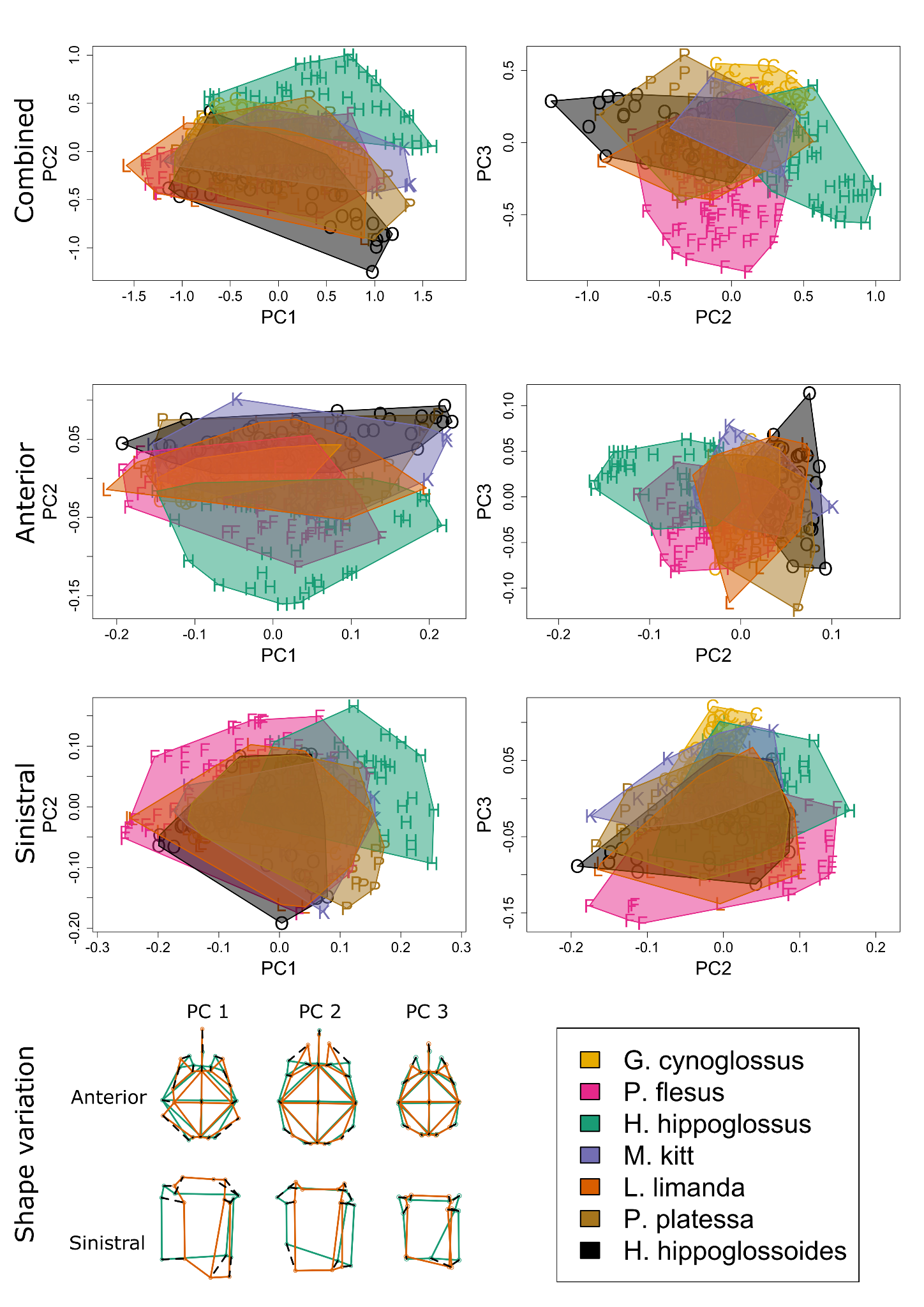


Figure S17. PCA and deformation grids of the caudal vertebra of Pleuronectidae for the different views.


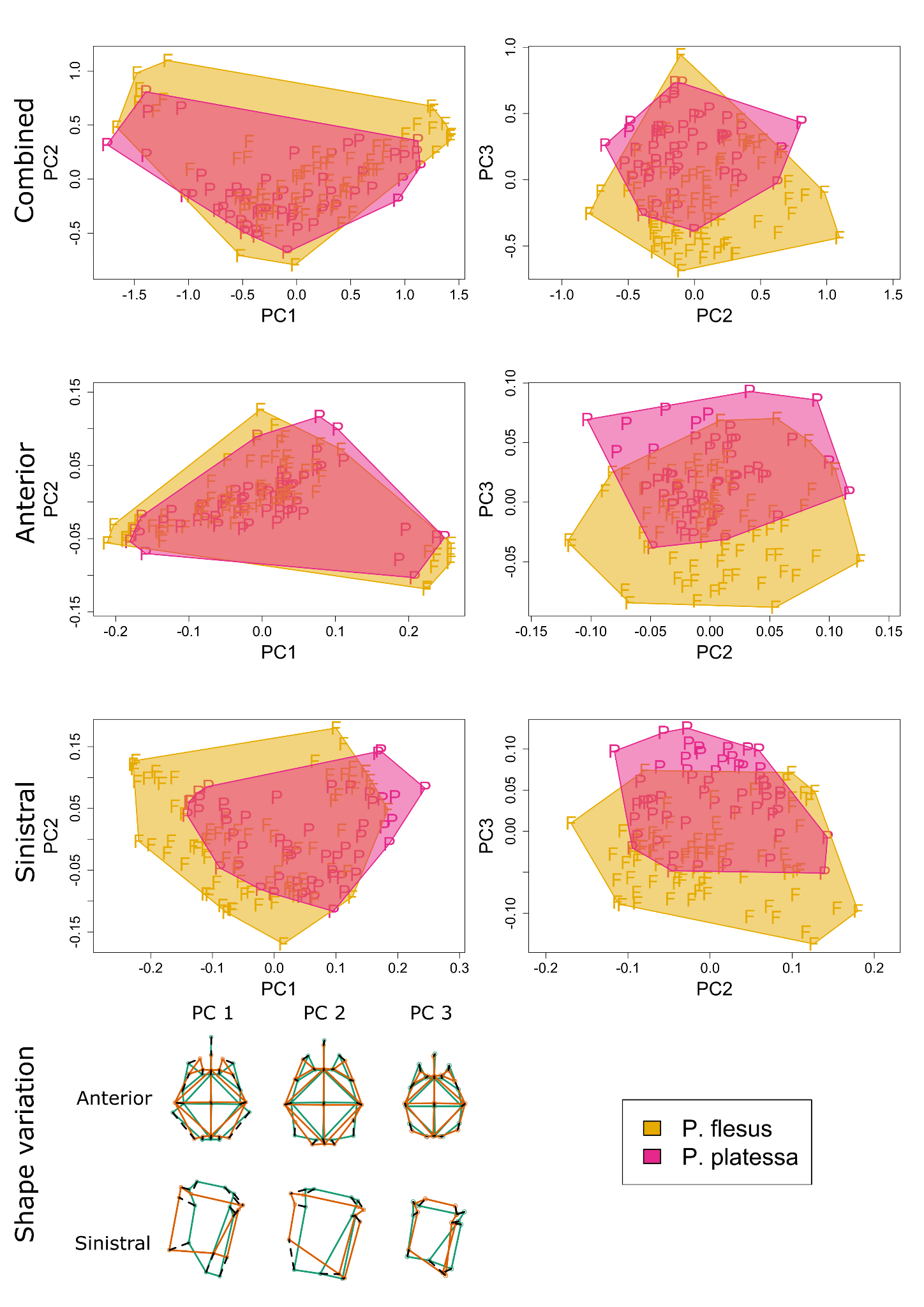


Figure S18. PCA and deformation grids of the caudal vertebra of plaice and flounder for the different views.


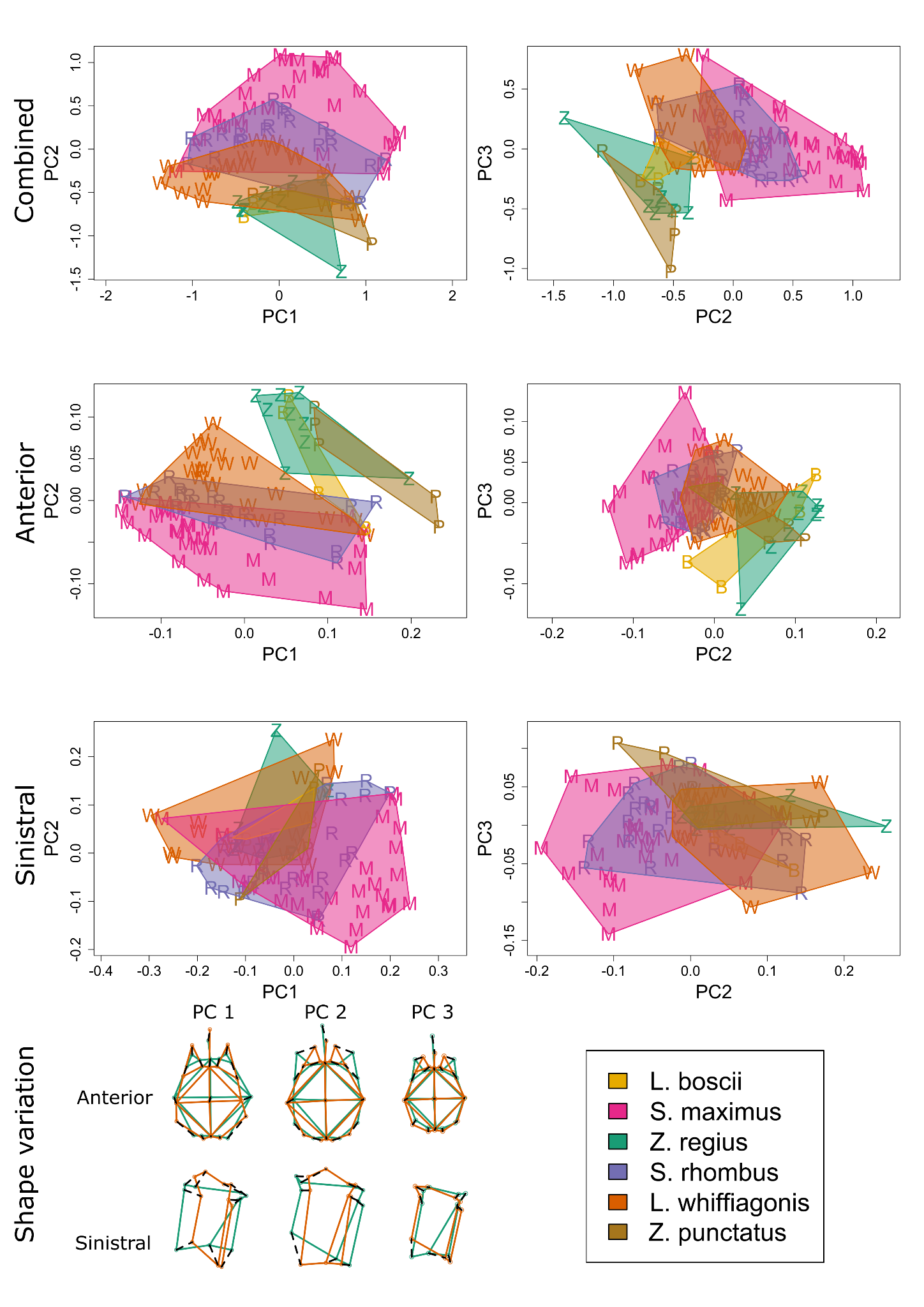


Figure S19. PCA and deformation grids of the caudal vertebra of Scophthalmidae for the different views.


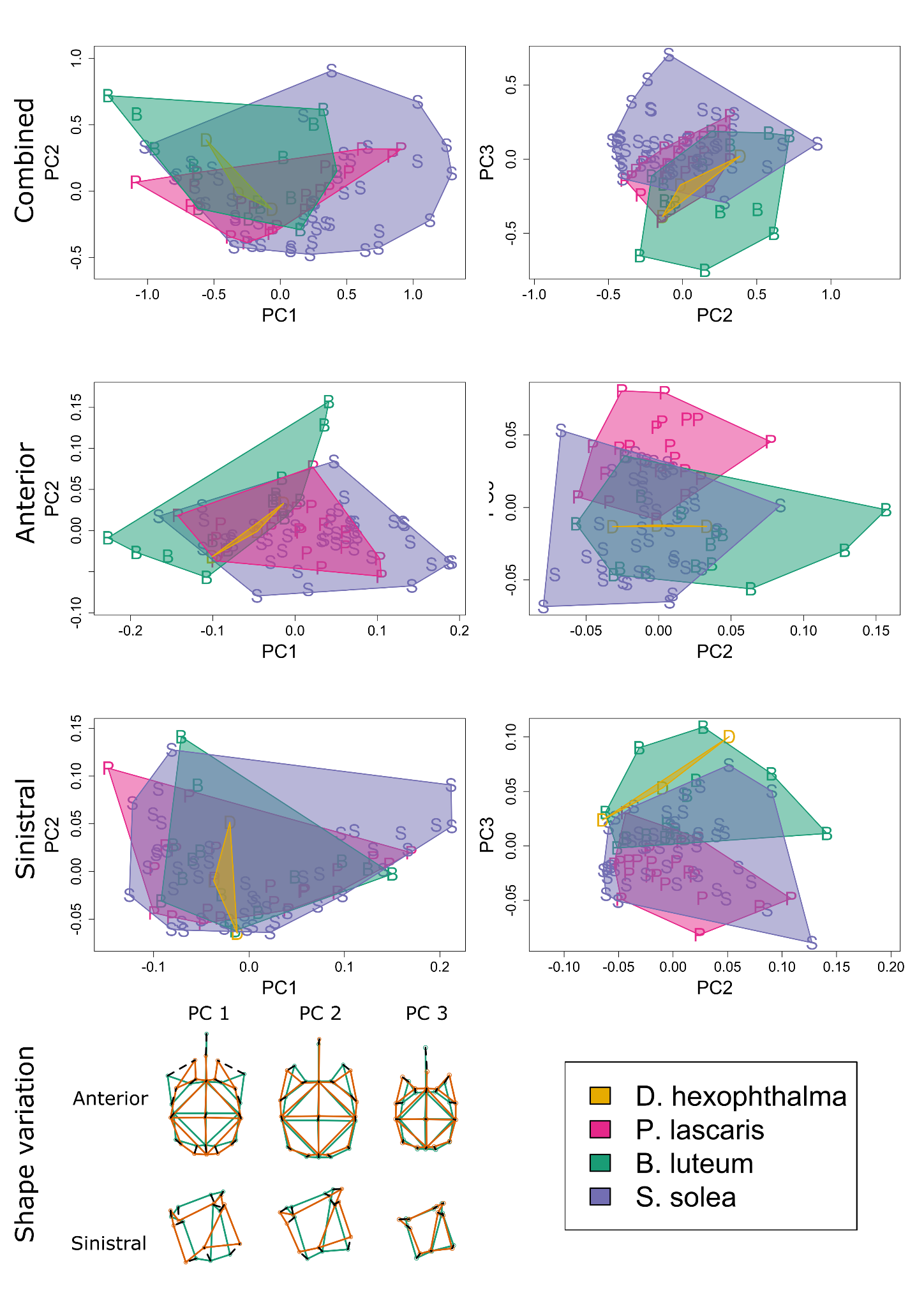


Figure S20. PCA and deformation grids of the caudal vertebra of Soleidae for the different views.

In the atlas, the landmark configuration including the arch tips (LM 1 to 13 anterior view and 1 to 9 sinistral view) was checked against the landmark configuration without the arch tip (LM 2 to 13 anterior view and 2 to 9 sinistral view) to see if the separation between taxa was better with or without the top of the neural arch. It seems that the presence of the tip of the neural arch can slightly improve the separation on the PCA plots between families (Figure S21), especially in the sinistral view, but within families the change seems minimal (Figure S22-24). Within Pleuronectidae, the separation is not improved (Figure S22).

For the precaudal vertebrae (LM 1 to 19 anterior view and 1 to 12 sinistral view versus 4 to 19 anterior view and 3 to 12 sinistral view), the loss of the arch tips also did not provide a large change in separation between taxa, and it even improved the separation between taxa in the sinistral view in some cases. In the caudal vertebra, the loss of the arch tips improved the separation between families and between species in the Scophthalmidae and Pleuronectidae families.

This indicates that the loss of the neural and haemal arches only results in minimal change regarding the separation between taxa. It was therefore decided to not further include the arch tips in the rest of the analysis (LM 1 in anterior view and LM 1 in sinistral view for the atlas and LM 1, 2, and 3 in anterior view and LM 1 and 2 in sinistral view for other vertebrae).


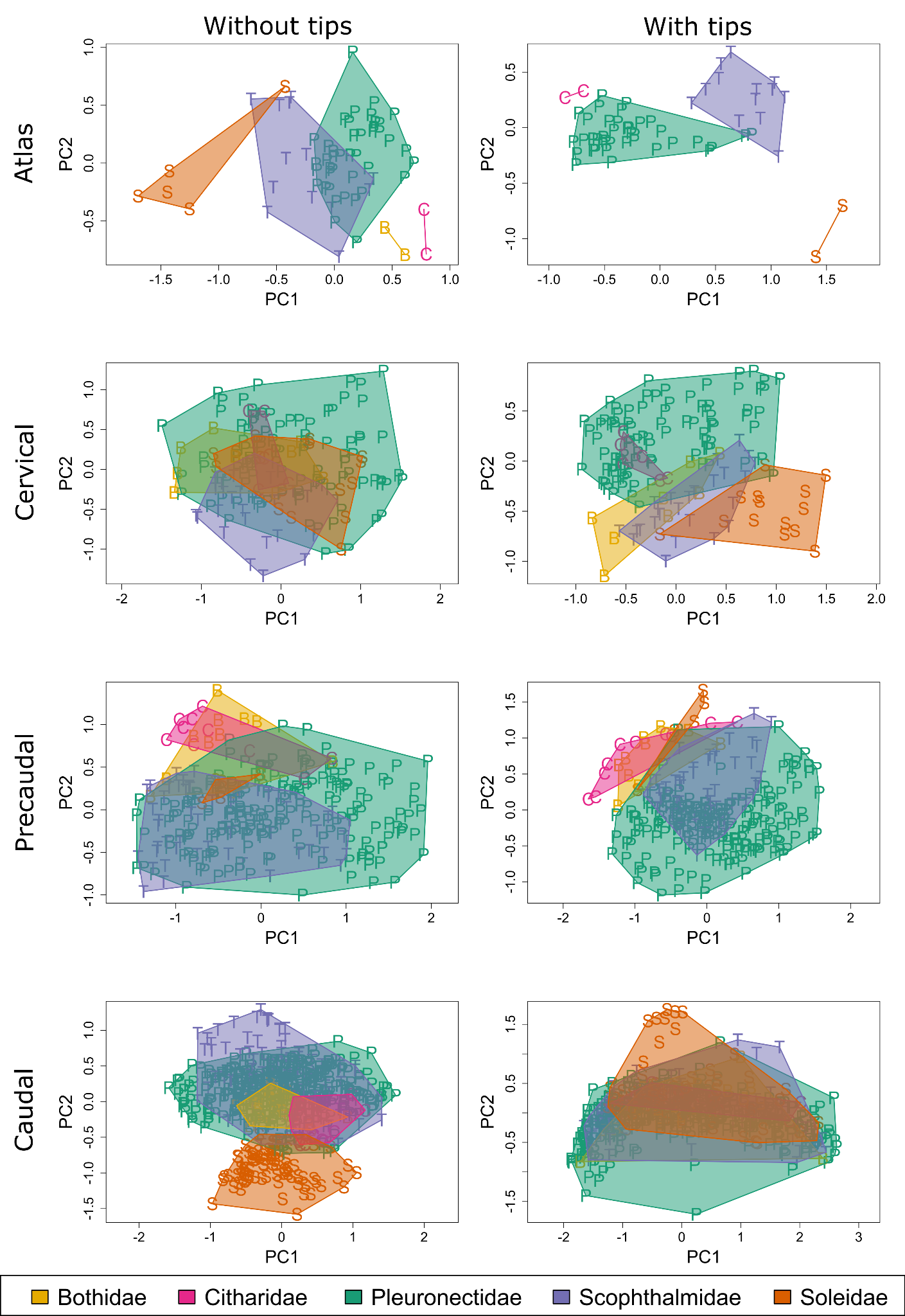


Figure S21. PCA plots for the vertebrae types per family comparing the landmark sets with or without the neural and haemal arch tips.


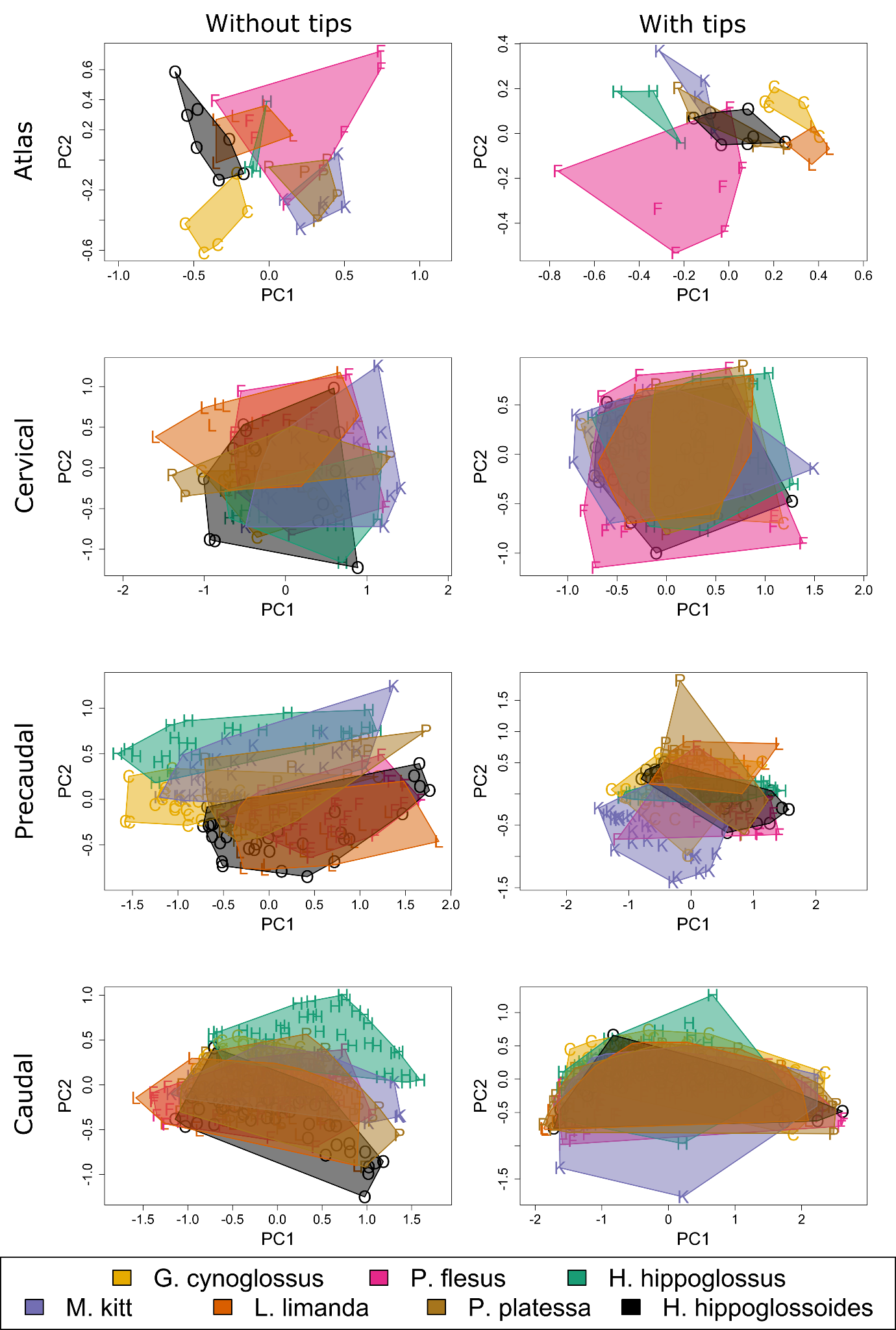


Figure S22. PCA plots for the vertebrae types for Pleuronectidae comparing the landmark sets with or without the neural and haemal arch tips.


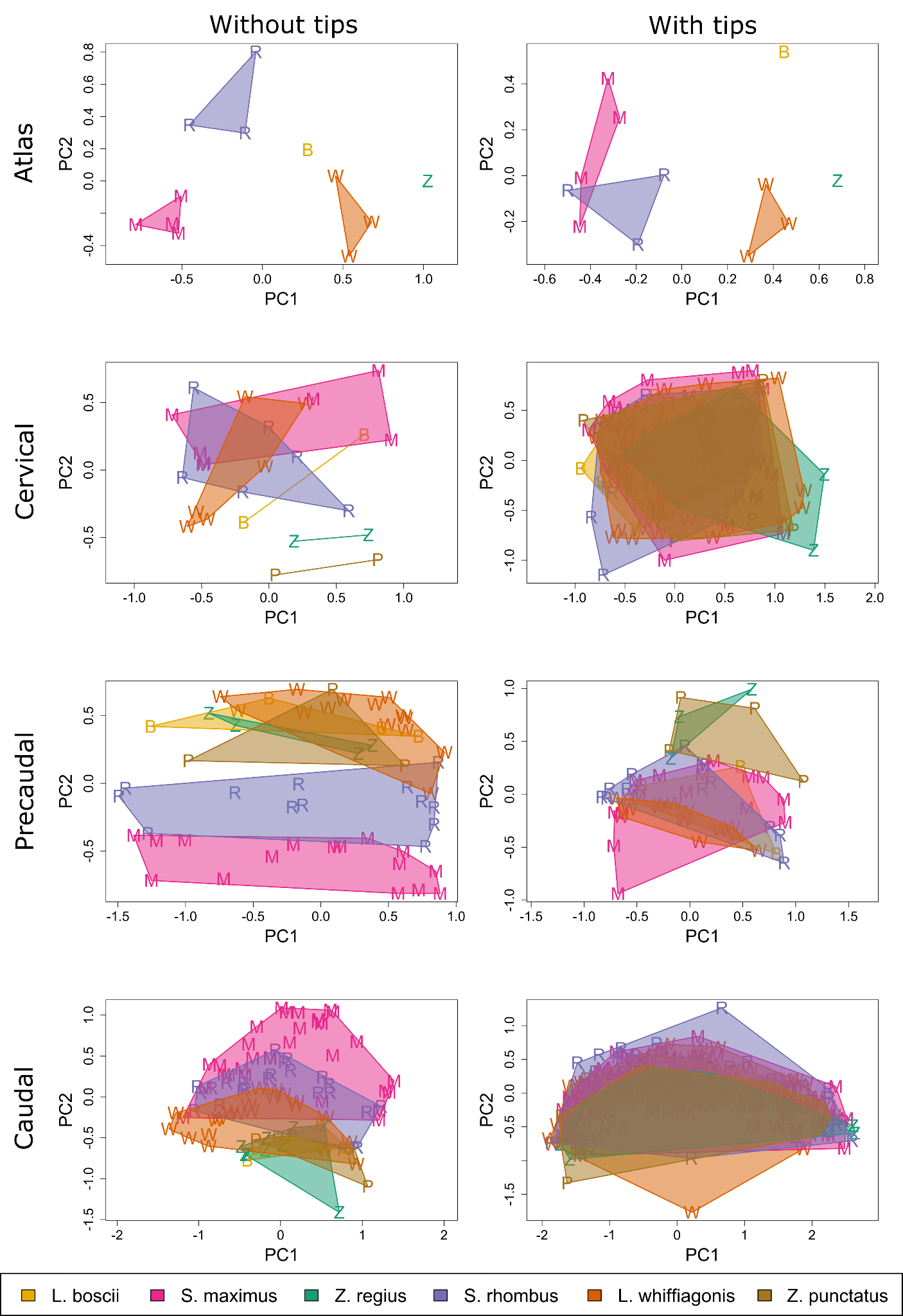


Figure S23. PCA plots for the vertebrae types for Scophthalmidae comparing the landmark sets with or without the neural and haemal arch tips.


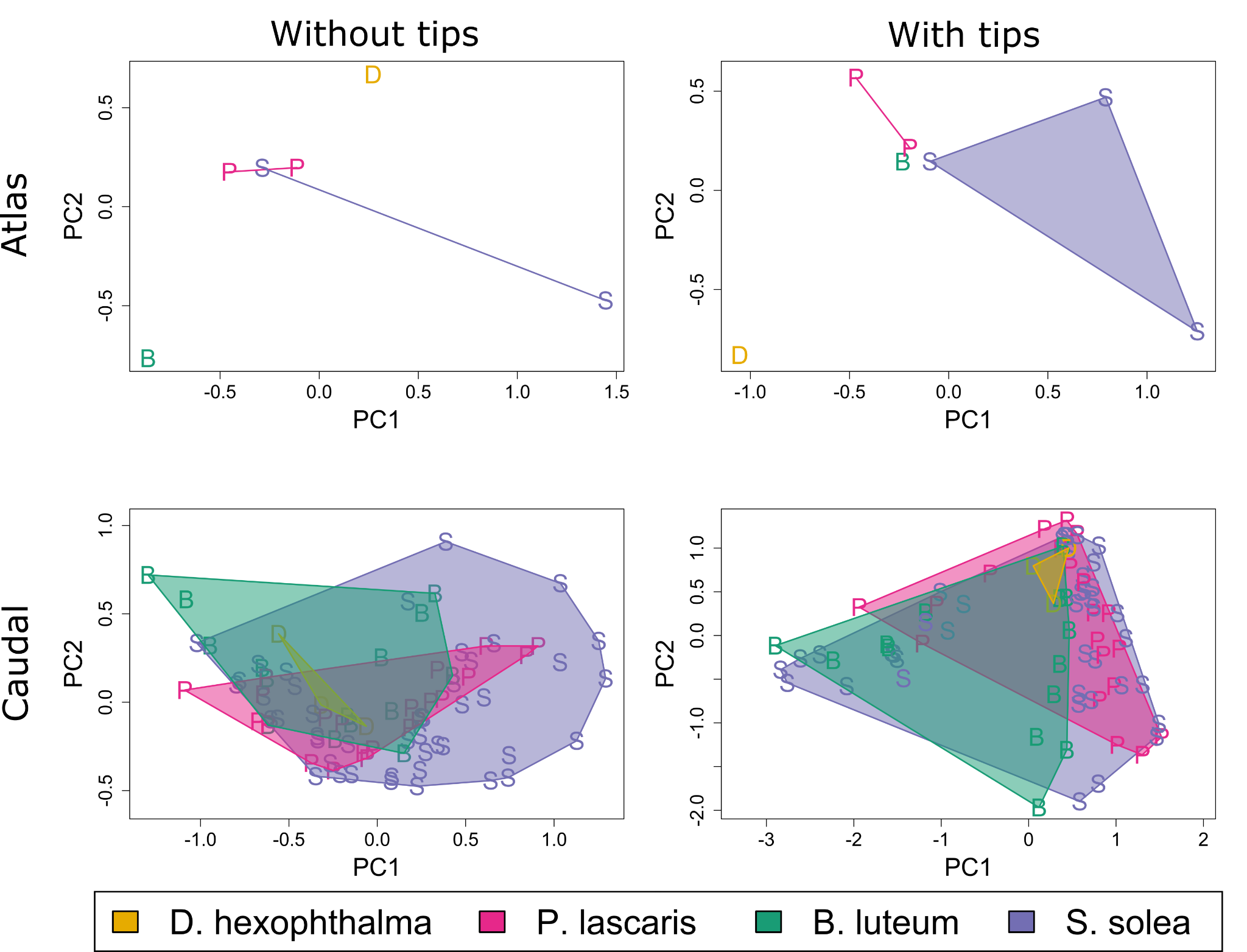


Figure S24. PCA plots for the vertebrae types for Soleidae comparing the landmark sets with or without the neural and haemal arch tips.


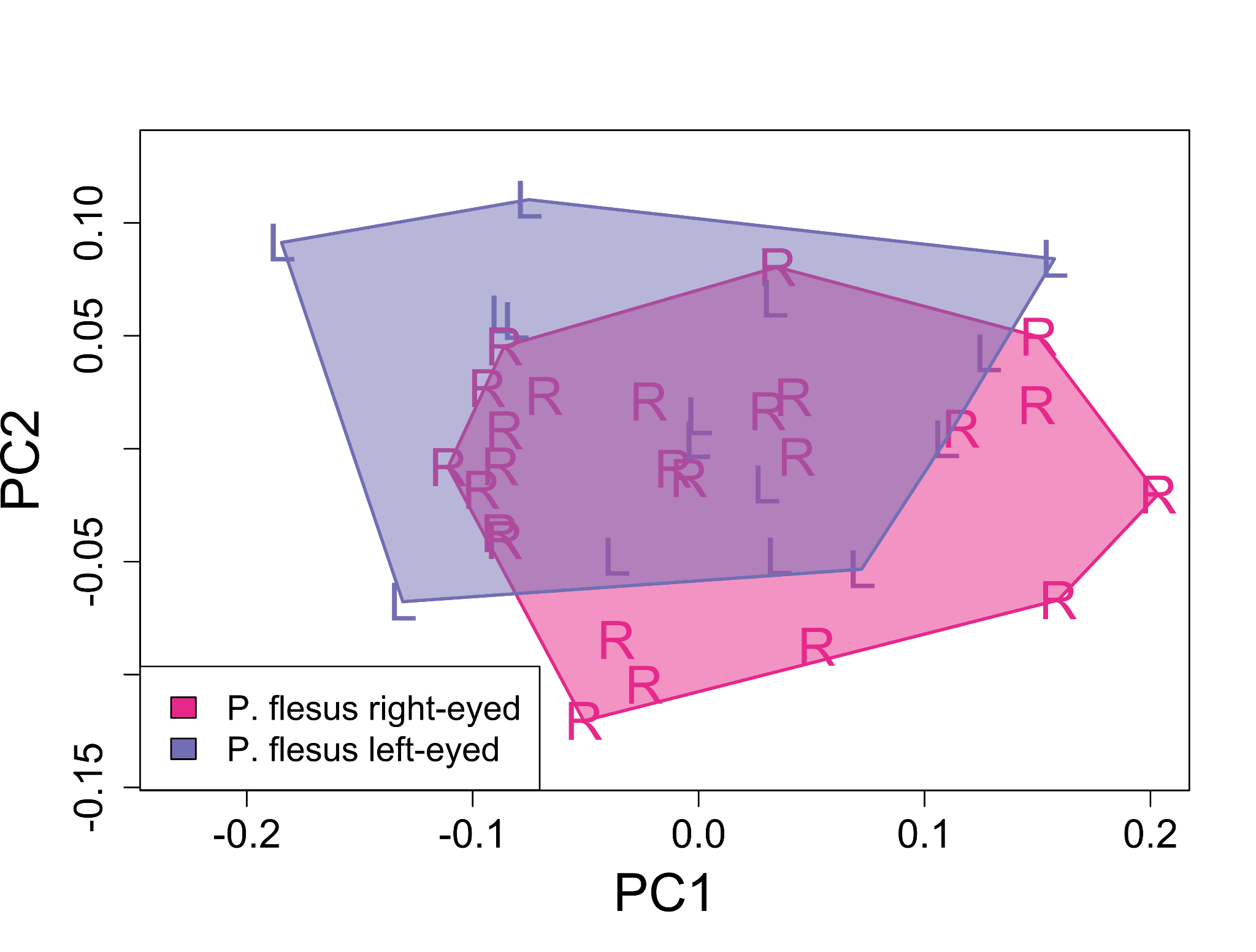


Figure S25. PCA plot of the precaudal vertebra of right-eyed and left-eyed *P. flesus* in anterior view.


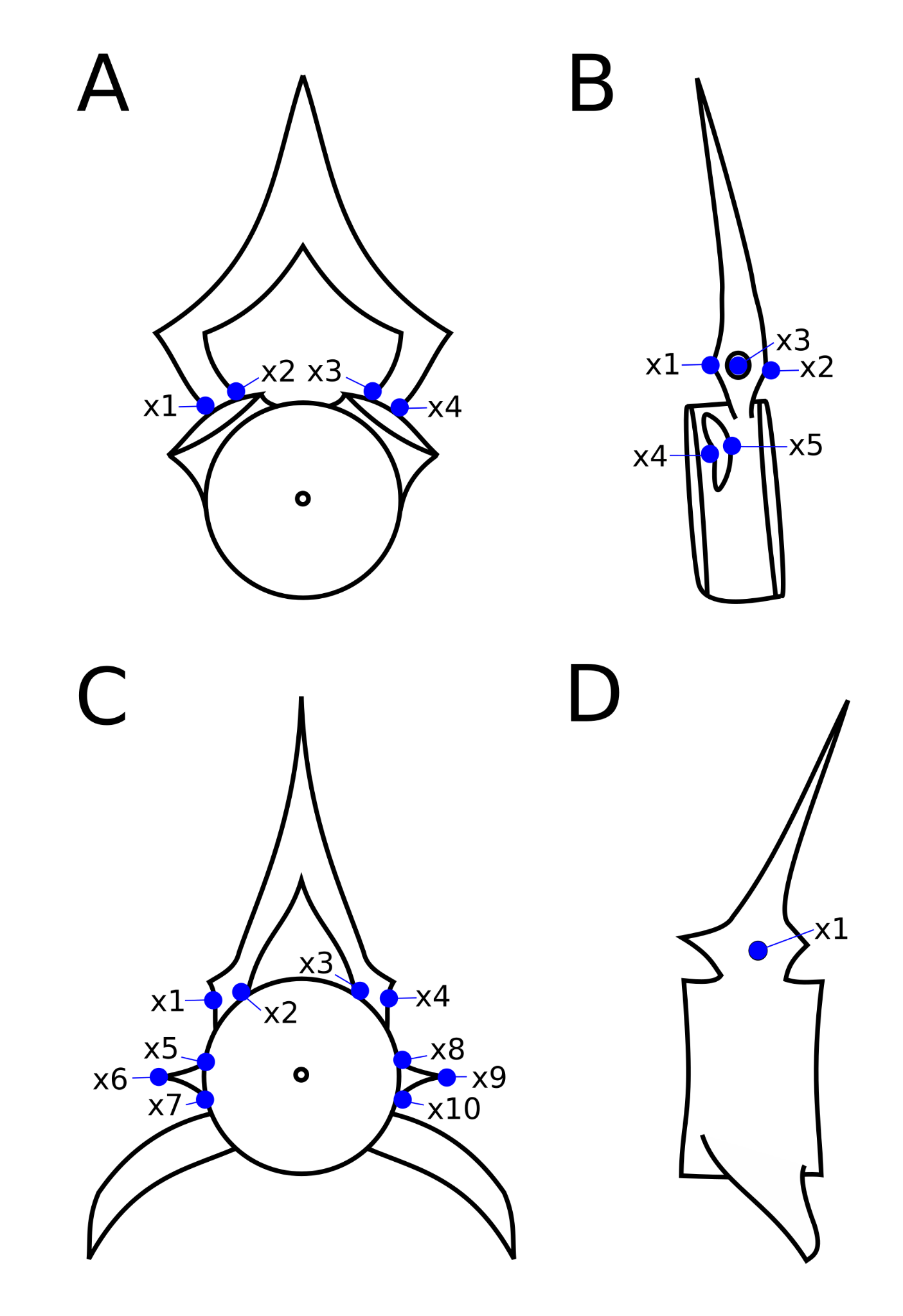


Figure S26. Landmark configurations of unusable landmarks not suggested for analysis as these are difficult to locate or their morphology is highly variable. The description of these landmarks can be found in table S2. A. anterior view of atlas vertebra; B. sinistral view of atlas vertebra; C. anterior view of cervical, precaudal, and caudal vertebrae; D. sinistral view of cervical, precaudal, and caudal vertebrae.
